# Molecularly Mediated Assembly of Metal Nanoparticles With Multifunctional Interfaces for Selective Detection of Small Gas Molecules

**DOI:** 10.1002/smll.74676

**Published:** 2026-07-23

**Authors:** Han‐Wen Cheng, Jin Luo, Xiajing Shi, Guojun Shang, Dong Dinh, Lidia Gebre, Zeqi Li, Seyed Danial Mousavi, Zakiya Skeete, Ke Pei, Wenbin You, Susan Lu, Tony T. Yuan, Chuan‐Jian Zhong

**Affiliations:** ^1^ Laboratory of Advanced Materials Department of Materials Science, College of Smart Materials and Future Energy Fudan University Shanghai China; ^2^ Department of Chemistry State University of New York at Binghamton Binghamton New York USA; ^3^ Systems Science and Industrial Engineering State University of New York at Binghamton Binghamton New York USA; ^4^ Center For Biotechnology (4D Bio^3^), Department of Radiology & Bioengineering Uniformed Services University of Health Sciences Bethesda Maryland USA

**Keywords:** chemiresistive sensing, gas detection, molecularly mediated assembly, multifunctional nanoparticle interface, nanostructured thin film, sensor array

## Abstract

The rational engineering of nanostructured interfaces with tunable molecular functionality is central to advancing chemiresistive sensing materials. Here, we report a programmable sensing platform based on the molecularly mediated assembly of metal–nanoparticle thin films. Multifunctional ligands bridge nanoparticles into cohesive networks with tunable interparticle nanogaps that act as active sensing hubs. This architecture exploits synergistic interfacial interactions, wherein metal‐site adsorption governs selective gas recognition, ligand‐mediated specific or ionic interactions modulate local dielectric properties, and matrix partitioning controls background vapor uptake, collectively regulating charge transport through the film. The resulting sensing films exhibit high selectivity toward small gas molecules (CO, NH_3_, and H_2_O) with well‐defined, thermally activated chemiresistive behavior. Systematic studies of binary and ternary gas mixtures reveal predictable, additive response patterns and robust analyte discrimination using principal component analysis. These results establish a mechanistically guided framework for molecularly assembled nanoparticle chemiresistors, linking metal composition, ligand chemistry, and film architecture to addressable interaction pathways, for constructing programmable chemiresistive sensor arrays capable of detecting small gas molecules.

## Graphical abstract

This work demonstrates a conceptual framework of three types of cooperative interaction pathways in molecularly assembled nanoparticle chemiresistors. Their relative contributions depend on nanoparticle composition, linker chemistry, film architecture, and analyte identity, modulating the effective tunneling barrier (Φ), interparticle spacing (*d*), and local dielectric environment (*ε*_eff_) in response to CO, NH_3_, and H_2_O probes.

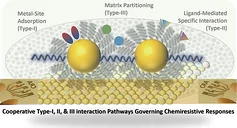

## 1 Introduction

Changes in electrical conductivity of nanomaterials arising from molecular adsorption have been exploited as chemiresistive sensing interfaces, which have applications ranging from environmental monitoring to medical diagnostics. Among the various nanomaterials, assemblies of metal nanoparticles represent an attractive class of chemiresistive materials because their electrical transport is dominated by interparticle electron tunneling, which exponentially depends on interparticle spacing, local dielectric environment, and electronic coupling. Subtle molecular interactions at the nanoparticle interface can generate pronounced and measurable electrical responses [1, 2, 3, 4]. Early studies of nanoparticle‐based chemiresistors largely relied on nonspecific gas/vapor partitioning into organic ligand shells [5, 6, 7]. This limited insight has restricted opportunities for rational control of sensitivity and selectivity. More recent work has begun to recognize the important roles of ligand structure, nanoparticle size, and nanoparticle composition in defining sensing behavior [8, 9, 10, 11]. However, a unifying framework that explicitly links molecular‐level interfacial interactions to charge‐transport modulation of chemiresistive responses remains underdeveloped. In this context, molecularly mediated assembly of metal nanoparticles provides a powerful strategy for engineering multifunctional sensing interfaces with controlled chemical and electronic properties. The thermally activated tunneling across ligand‐defined interparticle gaps makes the electrical response exquisitely sensitive to molecular adsorption events that alter tunneling barriers or local dielectric constants [12, 13, 14, 15].

**SCHEME 1:**
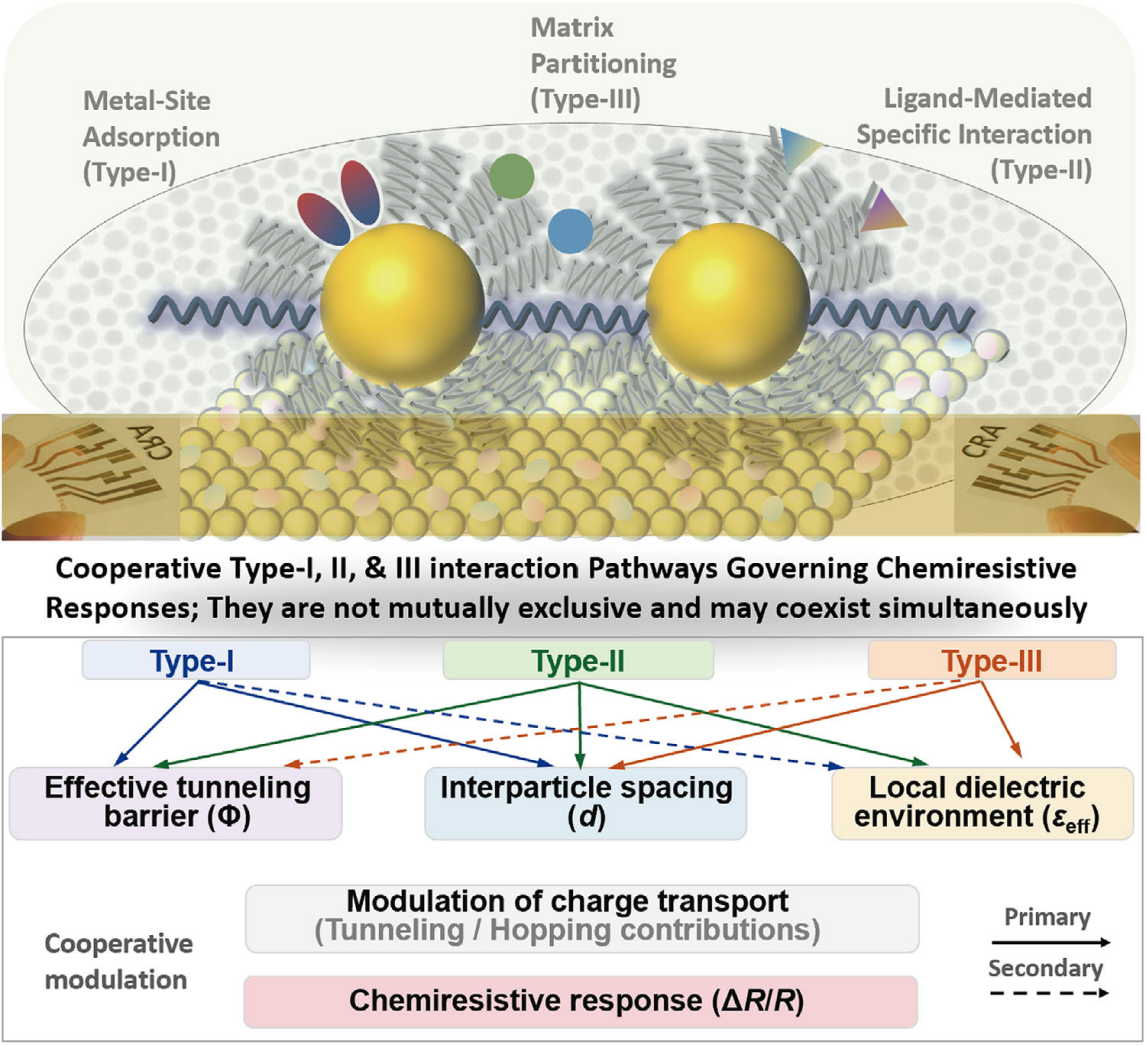
Conceptual framework illustrating cooperative Type‐I (metal‐site adsorption), Type‐II (ligand‐mediated specific interactions), and Type‐III (matrix partitioning) pathways in molecularly assembled nanoparticle chemiresistors. The three interaction classes are not mutually exclusive and may coexist simultaneously. Their relative contributions depend on nanoparticle composition, linker chemistry, film architecture, and analyte identity. Together, these interactions modulate the effective tunneling barrier (Φ), interparticle spacing (*d*), and local dielectric environment (*ε*_eff_), thereby altering charge transport and generating measurable chemiresistive responses (Δ*R*/*R*). CO, NH_3_, and H_2_O are used as representative probes because they preferentially emphasize Type‐I, II, and III interactions, respectively, while still permitting cooperative contributions from the remaining pathways.

We introduce herein a mechanistic framework that categorizes the interactions of nanoparticle‐assembled chemiresistors with adsorbed gas molecules into three distinct but coexisting types (Scheme 1). Type‐I interactions involve adsorption of gas molecules at electronically active metal sites, such as exposed nanoparticle facets, defect sites, or under‐coordinated atoms, where chemisorption or strong donor–acceptor binding directly perturb the electronic structure of the nanoparticle surface, leading to modulation of interparticle tunneling barriers. Type‐II interactions arise from specific, directional chemical interactions involving functional groups within the molecular linkers (e.g., hydrogen bonding, dipole–dipole interactions, or coordination to heteroatoms), which locally alter interparticle spacing or dielectric properties. Type‐III interactions correspond to partitioning or solvation of vapor molecules within the organic ligand matrix, resulting in bulk dielectric changes or film swelling that indirectly influence tunneling transport. Crucially, these interaction types are not mutually exclusive. Instead, their relative contributions can be rationally programmed by selecting nanoparticle composition and size (Type‐I), linker chemistry and functionality (Type‐II), and molecular architecture or film morphology (Type‐III), producing tunable chemiresistive responses to chemically similar gases (Scheme 1).

The framework is intended primarily for molecularly assembled nanoparticle systems operating in tunneling‐dominated transport regimes and is not proposed as a universal description of all chemiresistive sensing materials. Previous nanoparticle chemiresistors predominantly relied on nonspecific vapor uptake or single‐mode adsorption mechanisms, limiting selectivity and mechanistic interpretability [16, 17, 18], the importance of controlling adsorption energetics and interfacial electronic structure harnesses what is established in heterogeneous catalysis where ultrasmall metal and alloy nanoparticles exhibit size‐ and composition‐dependent binding energies and reactivity [19, 20, 21, 22, 23, 24]. Indeed, recent investigations of catalysts derived from ultrasmall alloy nanoparticles reveal high activity and durability for catalytic oxidation of CO in gas phase [25, 26]. Electrical conductivity in nanoparticle (NP) assemblies is governed by thermally‐activated electron tunneling between the metal nanoparticle cores. Gas adsorption influences interparticle coupling and charge transport [27, 28, 29], leading to adsorption‐induced modulation of interparticle tunneling and local dielectric environments. Such modulation, in combination with the usual VOC partitioning into the organic matrix to change the interparticle distance and dielectric properties [28], can effectively tune chemical sensing or catalysis [25, 26, 30, 31, 32].

In nanoparticle‐assembled chemiresistive films, charge transport occurs predominantly through thermally assisted electron tunneling across interparticle nanogaps. The effective tunneling barrier height (Φ) is determined by a combination of metal work function, ligand‐mediated dielectric environment, and adsorbate‐induced interfacial dipoles. Importantly, Φ is not identical to the apparent activation energy extracted from Arrhenius‐type resistance measurements, but rather constitutes its dominant physical contributor, such that small perturbations in Φ lead to amplified changes in the measured activation energy and macroscopic resistance.

The molecularly mediated nanoparticle assemblies investigated herein are built upon a platform that has been developed and quantitatively characterized over more than two decades. Previous studies established the synthesis of size‐controlled metal nanoparticles, ligand‐exchange and interparticle linking chemistries, thin‐film assembly mechanisms, interparticle spacing and morphology control, electrical transport characteristics, sensing responses, pattern‐recognition methodologies, and device stability (Table S1). In particular, quantitative relationships among linker chemistry, film structure, interparticle spacing, conductivity, and sensing behavior have been reported for a broad range of nanoparticle assembly systems (Table S1). The present work therefore focuses specifically on establishing a unified mechanistic framework that links these structural attributes to programmable chemiresistive responses through Type‐I (metal‐site adsorption), Type‐II (ligand‐mediated interactions), and Type‐III (matrix partitioning) pathways.

Inspired by these insights, we demonstrate that deliberate control of nanoparticle surface chemistry and ligand environment can be used to encode chemically rich information into electrical response patterns. In this sense, molecularly assembled nanoparticle chemiresistors bridge concepts from catalysis, materials chemistry, and electronic device physics. We systematically investigate molecularly‐mediated assemblies of metal nanoparticles with multifunctional interfacial sites for selective detection of small gas molecules such as CO and ammonia, and establish clear structure–property–function relationships governing sensitivity, selectivity, and response polarity. The results demonstrate that rational interface design, guided by the Type‐I/II/III interaction framework, enables chemiresistive sensing beyond empirical optimization, providing a versatile and mechanistically grounded platform for advanced gas detection. CO, NH_3_, and H_2_O were selected as model analytes because they span weakly chemisorbing, strongly Lewis‐basic, and highly polar vapor classes, respectively, providing a systematic means to probe how distinct interaction pathways govern chemiresistive responses across increasing chemical complexity.

## 2 Results and Discussion

For clarity, the molecularly assembled sensing films investigated in this work are designated according to the nanoparticle composition and linker chemistry. Au_2nm_ and Cu_5nm_ denote approximately 2‐nm gold nanoparticles and 5‐nm copper nanoparticles, respectively. PDT (1,5‐pentanedithiol), NDT (1,9‐nonanedithiol), DDT (1,12‐dodecanedithiol), ODT (1,8‐octanedithiol), MUA (11‐mercaptoundecanoic acid), and HDA (1,6‐hexanediamine) serve as representative linker molecules that create distinct interparticle architectures and interfacial environments. These molecularly engineered assemblies provide a versatile platform for systematically examining how nanoparticle composition, linker functionality, and film structure influence Type‐I, II, and III sensing interactions.

### 2.1 Site‐Specific Chemiresistive Responses to CO Exposures

**FIGURE 1:**
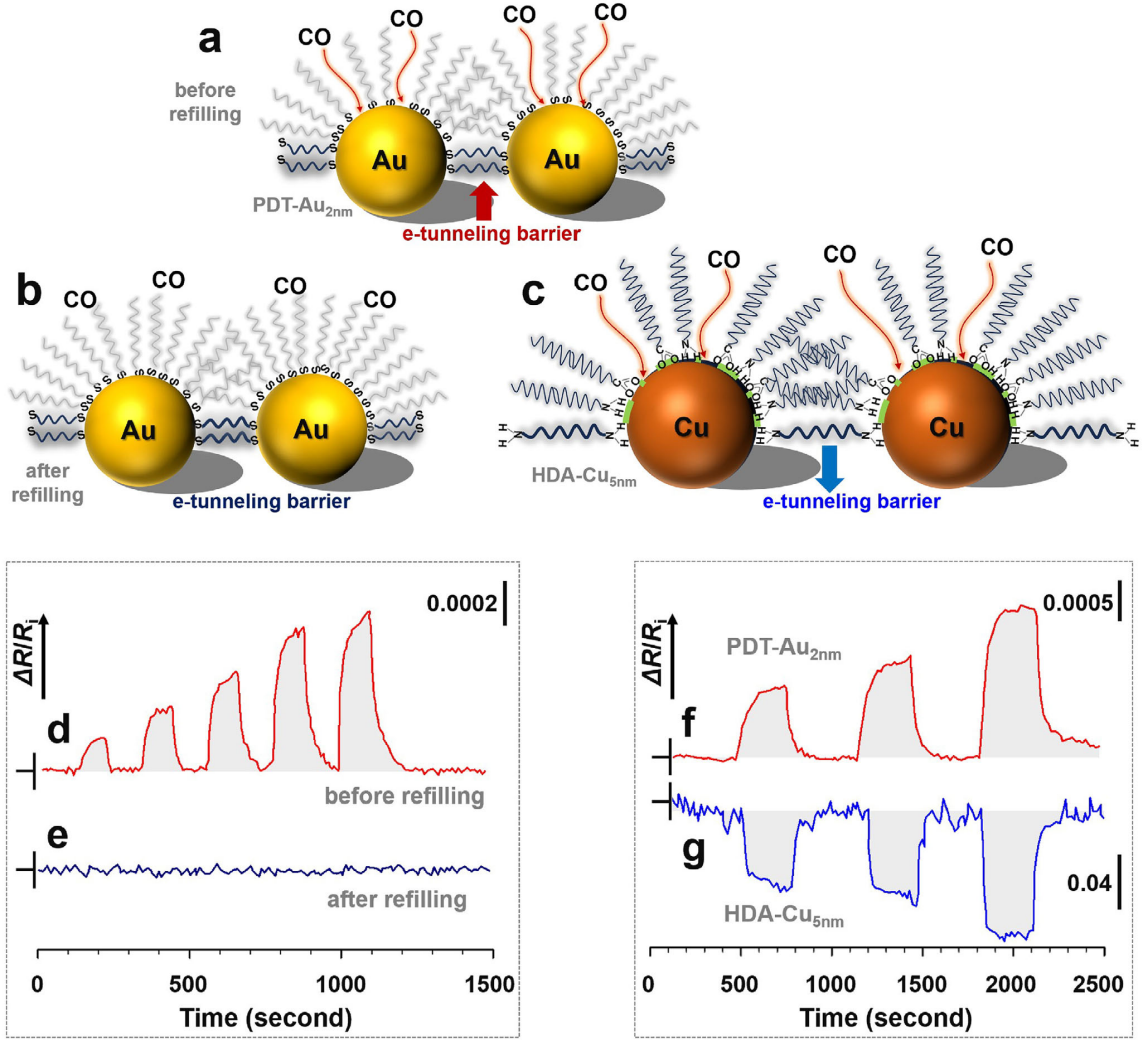
Chemiresistive responses to CO in molecularly mediated nanoparticle assemblies. CO response profiles (relative resistance change (Δ*R*/*R*_i_) vs. time) comparing PDT‐Au_2nm_ sensing films before (a,d) and after refilling (b,e), and PDT‐Au_2nm_ (a,f) and HDA‐Cu_5nm_ (c,g) sensing films (HDA linked Cu_5nm_ NPs capped with oleylamine (OAM) and oleic acid (OAC) mixed monolayer). (d–e) CO response profiles upon exposures to CO (67, 113, 159, 205, and 251 ppm in N_2_) for PDT‐Au_2nm_ sensing films before (d) and after (e) “refilling” capping/linking agents in the thin film. (f,g) CO response profiles (408, 502, and 596 ppm in N_2_) for PDT‐Au_2nm_ (f) and HDA‐Cu_5nm_ (g) sensing thin films. Mechanistic origin of chemiresistive responses: (a–c) Schematics of interfacial binding sites. (a,d) PDT‐Au_2nm_ exhibits a positive resistance change due to CO adsorption at electronically active Type‐I sites associated with exposed Au, which perturb interparticle electron tunneling barriers. (b,e) Refilling‐induced passivation of Au sites suppresses the CO response, confirming effective blocking of Type‐I interactions while Type‐III vapor partitioning remains accessible. (f) The positive response of PDT‐Au_2nm_ reflects weak Au–CO interactions that increase tunneling barriers through adsorption‐induced electronic perturbations. (g) In contrast, HDA‐Cu_5nm_ shows a negative resistance change, consistent with stronger CO–Cu chemisorption and redox interactions with surface oxide species that lower tunneling barriers and enhance charge transport. The results demonstrate that response polarity and sensitivity in nanoparticle‐assembled films are governed by metal identity and defect exposure. See Figure S1 for CO response sensitivities. Note that capping and linking molecules are shown on a portion of the nanoparticles and are not to scale, depicting defects in an idealized monolayer packing on the surface.

CO adsorption at exposed Au sites (Type‐I) through capping shell defects perturbs local charge density and alters tunneling barriers between nanoparticles, leading to an increase in resistance. Refilling the capping shell increases ligand packing density, reduces metal exposure, suppresses metal–CO interactions, but does not eliminate Type‐III (partitioning‐based) interactions. This concept is illustrated schematically in Figure 1a–c and experimentally demonstrated by the chemiresistive responses shown in Figure 1d–g. DT‐capped gold NPs (2 nm diameter, Au_2nm_) are assembled as chemiresistive sensing interfaces using PDT linker (PDT‐Au_2nm_). In comparison with as‐prepared (Figure 1d), “refilling” the capping/linking layer on the NPs by adding additional PDT into the assembling solution after the film has already been formed (Figure 1e) allowed filling the defect sites, leading to a more compact linking/packing of the capping layer on the nanoparticles and thus diminished Type‐I sites. In contrast to the clear positive response profile (Δ*R*/*R*) for the as‐prepared PDT‐Au_2nm_ thin film (Figure 1d), the “refilled” shows effectively no response to CO (Figure 1e). This finding can be explained by the effective blocking of Type‐I sites as a result of a refilling‐led compact linking/packing nanostructure. For the PDT‐refilled films, conductivity remains measurable because the interconnected nanoparticle network responsible for charge transport remains intact despite increased molecular coverage and reduced accessibility of exposed Type‐I sites. The suppression of CO response after refilling is consistent with the effective passivation of exposed Au defect sites that otherwise serve as catalytically active Type‐I binding sites. It does not prevent non‐specific VOC partitioning into the organic matrix, explaining the preserved responses to VOC such as hexane, benzene, and toluene.

Au–CO interactions involve relatively weak σ‐donation/π‐backdonation, which increase interparticle tunneling barriers and thus chemiresistive resistance, whereas CO chemisorption on Cu is stronger and modifies surface electronic states differently, leading to a resistance decrease. This contrast is illustrated in Figure 1f,g, comparing CO responses of PDT‐Au_2nm_ films with exposed Au defect sites (Figure 1a,f) and HDA‐Cu_5nm_ films with exposed Cu defect sites (Figure 1c,g). PDT‐Au_2nm_ exhibits a positive response (increase in Δ*R*/*R*) upon CO exposure that recovers upon N_2_ purging, while HDA‐Cu_5nm_ shows a negative response (decrease in Δ*R*/*R*) with similar reversibility. These opposite response polarities arise from site‐specific CO adsorption on Type‐I metal sites. On Au, CO adsorption perturbs interparticle tunneling barriers, increasing resistance, whereas on Cu, stronger CO interactions induce charge‐transport modulation that lowers resistance. Within the thermally activated tunneling framework, the positive response of Au films can be attributed to CO‐induced electron scattering, work‐function increase via dipole formation, or slight expansion of the capping layer that increases interparticle spacing. In contrast, the surface of Cu nanoparticles tends to form thin CuO/Cu_2_O shells, which interact with CO through surface reduction, electron injection, or removal of oxygen species, collectively lowering the effective tunneling barrier between Cu cores. The larger fluctuations in the HDA‐Cu_5nm_ response profiles than those observed for the DT‐Au_2nm_ films is primarily attributable to the substantially higher baseline resistance of the HDA‐Cu5nm assemblies, which arises from the use of the longer HDA linker molecules. This provides improved stabilization of the Cu nanoparticle surfaces against deep oxidation for the as‐prepared NPs in air but leads to increased electrical resistance of the film, resulting in a necessary tradeoff between surface protection and electrical conductivity. Despite the visually larger fluctuations, the observed signal amplitudes remain substantially larger than the baseline fluctuations, resulting in a high effective signal‐to‐noise ratio.

While Cu nanoparticles were linked using HDA, it allowed us to assess the role of catalytic surface sites of Cu NPs, which was not intended to make a direct comparison of these two device types within the framework of Type‐I/II/III interactions. Mechanistically, electron transfer to adsorbed CO on Au nanoparticles (Au^δ+^–CO) perturbs the electronic structure of the metallic cores, increasing tunneling resistance. For Cu nanoparticles, surface oxidation produces a hole‐accumulation layer that enhances conductivity; however, in ultrathin Cu_x_O shells, electron tunneling dominates charge transport. In this regime, CO adsorption or reaction with surface oxygen injects electrons into the n‐type Cu_x_O shell, reducing the tunneling barrier height and exponentially enhancing interparticle electron transport. Consequently, CO exposure decreases resistance in Cu‐based assemblies, opposite to the response observed for Au films. The strong sensitivity of tunneling to barrier height enables large resistance changes even at low CO concentrations. This interpretation is supported by Langmuir adsorption modeling of the response profiles [33] (Figure S2), where allows extraction of the apparent adsorption rate constant (*k*_a_). PDT‐Au_2nm_ exhibiting *k*_a_ = 5×10^−4^ s^−1^ at low CO concentrations (< 200 ppm) and a reduced value at higher concentrations (5×10^−5^ s^−1^) due to adsorbate–adsorbate interactions, whereas HDA‐Cu_5nm_ shows a higher *k*_a_ (1×10^−4^ s^−1^) at elevated concentrations, consistent with stronger CO adsorption on Cu sites.

**FIGURE 2:**
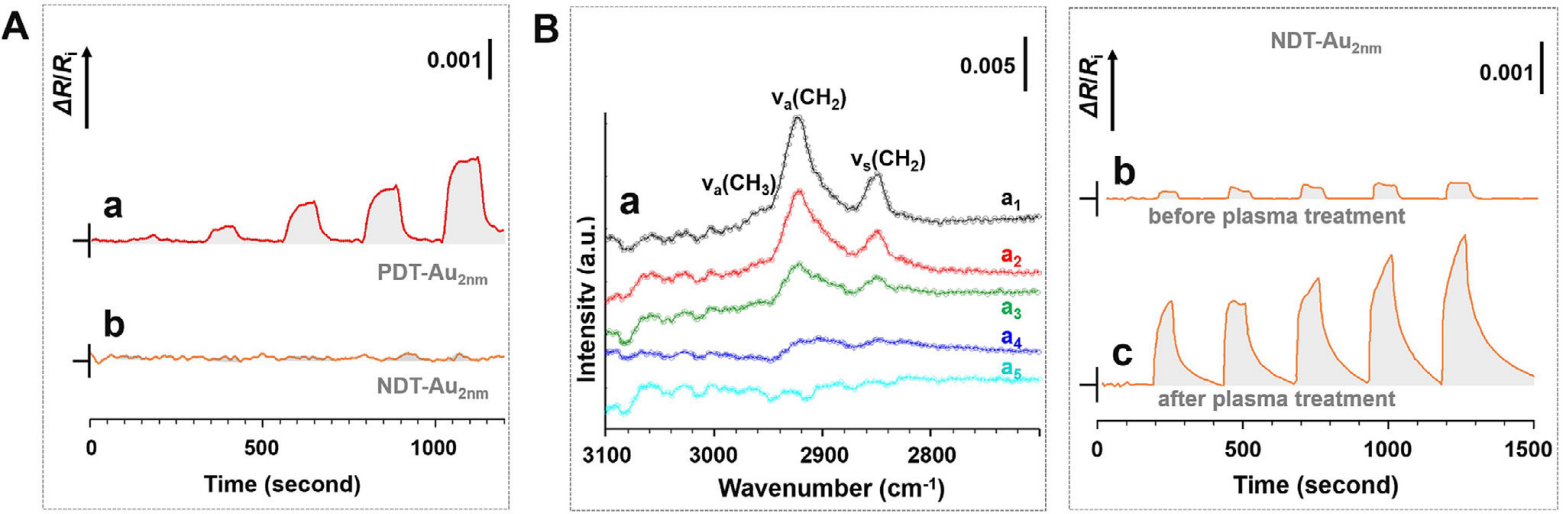
Linker chain length‐dependent and plasma‐induced Type‐I metal‐site interactions. (A) Linker chain length–dependent regulation of defect density. Response profiles to CO (136, 251, 366, 481, and 596 ppm in N_2_) for PDT‐Au2nm (a) and NDT‐Au_2nm_ (b) thin films on IME devices. IME device: finger space 5 µm, finger width 5 µm, finger length 100 µm. Au_2nm_: 2‐nm sized Au NPs. The results illustrate how linker chemistry modulates defect formation and indirectly controls Type‐I metal‐site accessibility for CO adsorption. (B) (a) FTIR spectra for NDT‐Au_2nm_ sensing film after oxygen‐plasma treatment for varying durations: 0 (a_1_), 1 (a_2_), 2 (a_3_), 6 (a_4_), and 12 (a_5_) min, showing progressive removal of alkyl thiolate ligands. (b,c) Response profiles to CO (66, 111, 156, 202, and 247 ppm in N_2_) for NDT‐Au_2nm_ sensing films before (b) and after (c) 1‐min plasma treatment (resistance of the thin films: b: 42.7 kΩ; c: 7.97 kΩ), demonstrating enhanced sensitivity arising from increased availability of Type‐I binding sites. *Note*: see Figure S3 for response sensitivities.

As shown in Figure 2A with α,ω‐difunctional alkyl dithiol linkers of two different chain lengths (PDT, and NDT), pronounced differences in CO response are observed. PDT‐Au_2nm_ assemblies exhibit a strong, linear increase in Δ*R*/*R* with CO concentration (Figures 2a; Figure S3a), whereas NDT‐Au_2nm_ assemblies show minimal response under identical conditions (Figures 2b; Figure S3b). These trends indicate a strong dependence of CO sensitivity on a combination of the linker‐mediated interfacial structure and differences in film thickness or Au nanoparticle loading. Shorter linkers such as PDT induce greater disruption of the dodecanethiol capping layer due to reduced chain–chain cohesive interactions, resulting in higher defect densities and increased accessibility of Au surface sites for CO adsorption. In contrast, NDT linkers closely match the length of the capping ligands, leading to more ordered monolayers with fewer defects and minimal CO response. While CO adsorption itself occurs primarily at Type‐I metal sites, the availability of these sites is indirectly regulated by Type‐II linker‐mediated effects. This assessment is further supported by fitting the response profiles (see Figure S4), the adsorption rate constant (*k*_a_) shows 1 × 10^−4^ s^−1^ for PDT‐Au_2nm_ at low concentration range (<200 ppm) (slightly larger than that in Figure 1d). In comparison, NDT‐Au_2nm_ shows a much greater adsorption rate constant (1 × 10^−3^ s^−1^) after plasma treatment, supporting the increased exposures of surface adsorption sites. Accordingly, changes in the fitted activation energy should be interpreted as reflecting adsorption‐induced modulation of the effective tunneling barrier height Φ, rather than a transition to a fundamentally different transport mechanism.

The role of exposed Type‐I sites is further examined by combining oxygen‐plasma treatment with spectroscopic and electrical characterization of NDT‐Au_2nm_ thin films (Figure 2B). Controlled plasma exposure was used to partially remove the capping/linking ligands, thereby increasing the availability of catalytically active Au surface sites. The extent of ligand removal was directly verified by FTIR spectroscopy (Figure 2Ba), which shows a progressive decrease in the characteristic methyl and methylene stretching bands, ν_a_,_s_(CH_3_) (2955 and 2872 cm^−1^) and ν_a_,_s_(CH_2_) (2917 and 2848 cm^−1^), with increasing plasma treatment time. The attenuation of these vibrational features confirms the plasma‐induced desorption of alkyl thiolate ligands, exposing Au surface sites. Consistent with ligand removal, plasma treatment results in a pronounced decrease in the electrical resistance of the sensing films, reflecting reduced interparticle tunneling barriers due to diminished organic spacer coverage. Correspondingly, the chemiresistive response to CO is significantly enhanced after plasma treatment (Figure 2Bc). The plasma‐treated NDT‐Au_2nm_ films exhibit a substantially larger resistance increase upon CO exposure than the untreated films, while responses to larger organic vapors remain comparatively modest. This selectivity indicates that small molecules such as CO can penetrate the residual ligand matrix and adsorb directly onto newly exposed Au sites. These observations are characteristic of strengthened Type‐I interactions, in which direct adsorption of CO at electronically active metal sites perturbs local charge density and increases the effective tunneling barrier between adjacent nanoparticles. The gradual loss of response after prolonged air exposure is attributed to passivation of exposed Au sites by oxygen‐containing species and potential nanoparticle aggregation or sintering, both of which reduce the density of active Type‐I sites. Figure 2B establishes that deliberate manipulation of metal site availability, validated spectroscopically, provides a direct route to tuning chemiresistive sensitivity through interfacial electronic effects.

**FIGURE 3:**
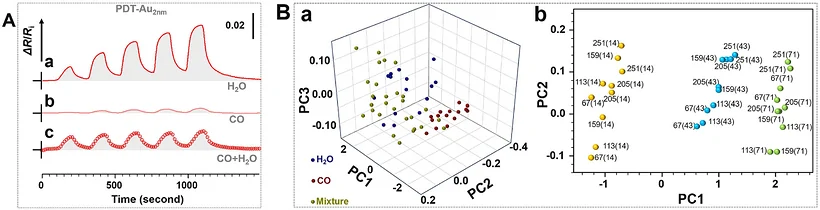
Cooperative interactions in gas mixtures. (A) Response profiles of PDT‐Au_2nm_ sensing film to H_2_O (a), CO (b), and a mixture of CO and H_2_O (c) with different concentrations: (a) H_2_O vapor (14, 28, 43, 57, 71 ppm in N_2_); (b) CO (67, 113, 159, 205, and 251 ppm in N_2_); (c) CO/H_2_O (67/14, 113/28, 159/43, 205/57, and 251/71 ppm in N_2_). The results highlight competitive and cooperative contributions from metal‐site adsorption (Type‐I) and vapor partitioning within the ligand matrix (Type‐III). *Note*: see Figure S5 for response sensitivities. (B) 3D and 2D PCA plots for CO, H_2_O, and mixture of CO and H_2_O using a sensor array (PDT‐, NDT‐, and DDT‐Au_2nm_). (a) PCA score plot of CO, H_2_O, and their mixture in 3D. (b) PCA score plot of mixture (CO/H_2_O) with different component concentration in the PC1‐PC2 plane. The data label in the figure means the concentration (ppm) of components in the test mixture. For example, 67(14) means 67 ppm CO and 14 ppm water in the mixture. The even distribution of points facilitates the determination of the component concentration.

As shown in Figure 3A, the response of nanoparticle‐assembled sensing films to CO, H_2_O vapor, and their mixtures to evaluate molecular recognition under chemically complex conditions are explored. For PDT‐Au_2nm_ films with controlled defect densities, exposure to CO produces a resistance increase characteristic of Type‐I interactions, while exposure to H_2_O yields a larger response dominated by Type‐III interactions, arising from partitioning of water molecules into the organic ligand matrix. When CO and H_2_O are introduced simultaneously, the resulting chemiresistive responses fall between those of the pure analytes and exhibit concentration‐dependent scaling (Figures 3A; Figure S5). At fixed concentrations of one component, the response varies linearly with the concentration of the other, indicating that the sensing signal reflects a combined contribution from both interaction modes. CO adsorption at exposed Au sites perturbs electronic coupling, while H_2_O partitioning modifies the dielectric environment and interparticle spacing within the ligand shell. These results demonstrate that the nanoparticle‐assembled films encode chemically specific information through cooperative Type‐I and III interactions, rather than responding solely to total vapor concentration. The ability to distinguish pure gases from mixtures highlights the potential of molecularly engineered nanoparticle interfaces for molecular recognition in complex gas environments. The above assessment is supported by fitting the response profiles (see Figure S6), yielding *k*_a_ ∼7 × 10^−4^ s^−1^ for H_2_O vapor only and 1 × 10^−4^ s^−1^ for CO only on PDT‐Au_2nm_. In comparison, the *k*_a_ (1 × 10^−4^ s^−1^) for CO+H_2_O mixture is the same as that for CO only. Even by plotting against H_2_O fraction, the *k*_a_ (4 × 10^−4^ s^−1^) remains half of that for H_2_O only. These results support the higher selectivity of the surface adsorption sites to CO in the presence of H_2_O.

To further establish the selectivity of the nanostructured sensing films toward chemically complex gas mixtures, multivariate pattern recognition techniques were applied to analyze the sensor array data. Both qualitative identification of analytes and quantitative estimation of mixture composition were examined. Principal component analysis (PCA) is employed here as a multivariate statistical tool to visualize and quantify response pattern separability, rather than as a predictive machine‐learning classifier. As illustrated in Figure 3Ba, PCA plot yields well‐separated clusters corresponding to CO, H_2_O, and their binary mixtures, demonstrating clear discrimination among individual analytes and mixed vapors. Prior to PCA analysis, the sensor‐response data were normalized and mean‐centered to minimize scale‐dependent contributions among the sensing elements. PCA is used herein as a multivariate visualization and separability tool rather than as a predictive machine‐learning classifier. Accordingly, the principal components are interpreted qualitatively to identify systematic variations in sensing behavior associated with different analytes and interaction pathways.

In addition, binary mixture patterns with different component concentrations are distinctly resolved in the principal component space (Figure 3Bb). At fixed CO/H_2_O ratios, the data points align along well‐defined linear manifolds, reflecting additive and independent contributions from Type‐I and III interactions. This behavior represents an ideal characteristic for concentration estimation in multicomponent gas sensing. Using the first three principal components (PC1, 2, and 3) as input features, discriminant analysis was further employed to evaluate recognition performance. The resulting recognition rates (Table S2) confirm effective identification of both individual analytes and mixture compositions, demonstrating the feasibility of multivariate analysis for enhancing selectivity and quantitative capability in nanoparticle‐based chemiresistive sensor arrays.

These results establish a unified mechanistic framework for chemiresistive sensing based on molecularly mediated nanoparticle assemblies by, (i) introducing the classification of gas–film interactions into Type‐I, II, and III modes and linking them to modulation of interparticle electron tunneling (Figure 1), (ii) demonstrating that plasma‐induced ligand removal enhances Type‐I metal‐site interactions and CO sensitivity, and that Type‐II linker chemistry governs defect formation and indirectly controls the availability of Type‐I binding sites (Figure 2), and (iii) showing how Type‐I and Type‐III interactions cooperate and compete in gas mixtures, enabling chemically specific response patterns (Figure 3). Chemiresistive sensing in nanoparticle‐assembled films can be rationally programmed through interfacial design, providing a mechanistically grounded strategy for selective gas detection beyond empirical optimization. These observations are consistent with the mechanistic framework illustrated in Scheme 1, in which CO adsorption at metal‐centered Type‐I sites modulates interparticle tunneling barriers, while ligand‐mediated Type‐II and matrix‐driven Type‐III interactions remain comparatively weak.

### 2.2 Responses to Exposures to NH_3_ and CO in Binary/Ternary Mixtures

**FIGURE 4:**
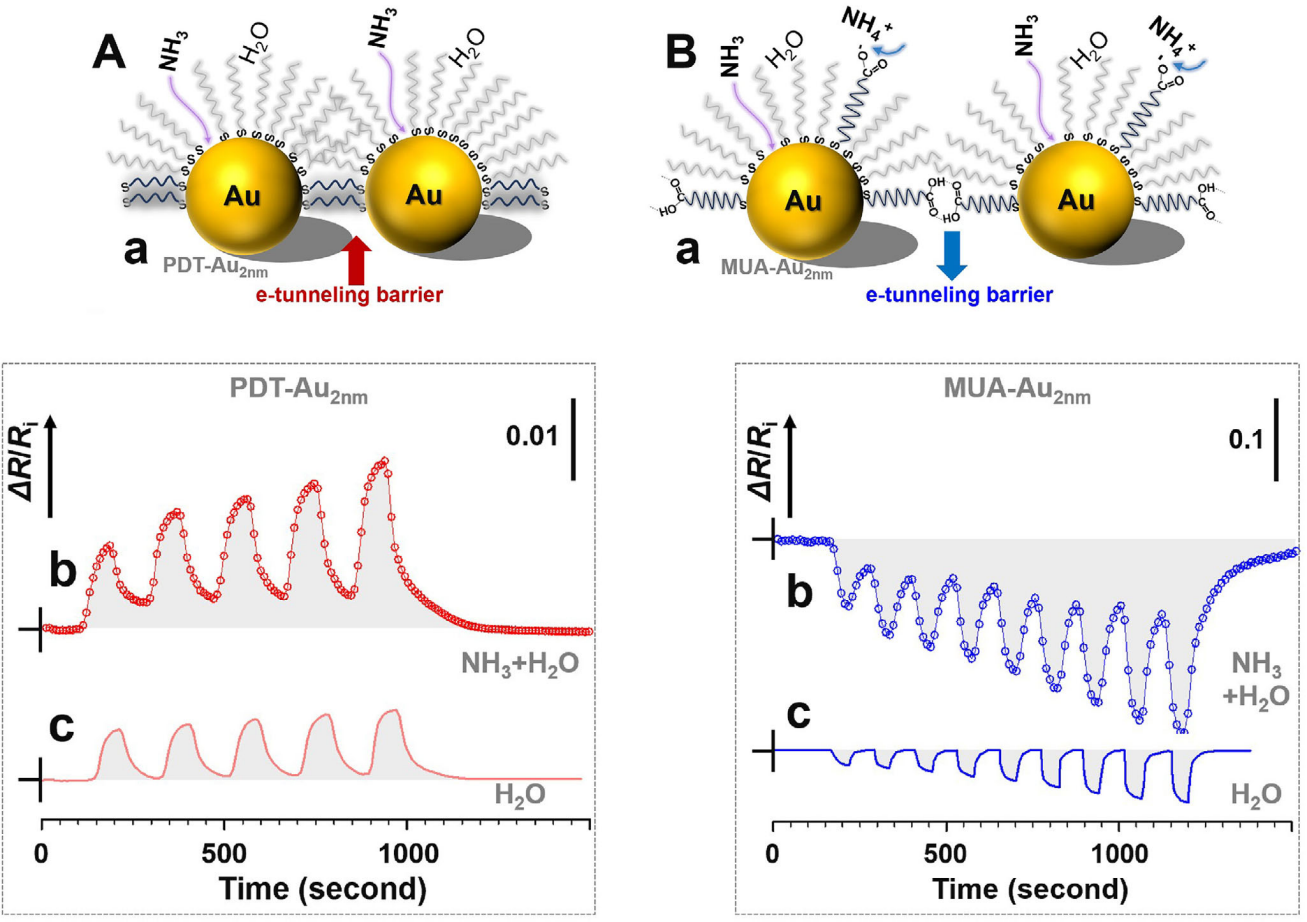
Chemiresistive responses of PDT‐ and MUA‐linked Au_2nm_ nanoparticle films to NH_3_ and NH_3_/H_2_O binary mixtures. (A) (a) Schematic illustration of the interfacial binding sites. (b, c) Response profiles to mixtures of ammonia and water vapor (b) and water vapor alone (c) with different concentrations: (b) NH_3_/H_2_O (223/102, 446/122, 669/142, 892/162, and 1115/182 ppm); (c) H_2_O vapor (102, 122, 142, 162, and 182 ppm) for the PDT‐Au_2nm_ sensing film. *Note*: see Figure S7 for response sensitivities. These interfaces are dominated by Type‐I interactions arising from NH_3_ adsorption at exposed metal sites, with additional modulation from Type‐III vapor partitioning within the ligand matrix. Differences in response magnitude reflect linker‐dependent variations in nanoparticle spacing and surface accessibility. (B) (a) Schematic illustration of the interfacial binding sites. (b, c) Response profiles to mixtures of ammonia and water vapor (b) and water vapor alone (c) with different concentrations: (b) NH_3_/H_2_O (124/122, 186/158, 248/194, 310/230, 372/266, 434/302, 496/338, 558/374, and 620/410 ppm); (c) H_2_O vapor (122, 158, 194, 230, 266, 302, 338, 374, and 410 ppm) for the MUA‐ linked Au_2nm_ sensing film. *Note*: see Figure S9 for response sensitivities. The negative resistance change is governed by Type‐II interactions involving acid–base reactions and formation of ammonium–carboxylate ion pairs, which increase the effective dielectric constant of the interparticle medium and dominate charge transport modulation. Note that capping and linking molecules are shown on a portion of the nanoparticles and are not to scale, depicting defects in an idealized monolayer packing on the surface.

To further elucidate how different binding interactions operate within the thin‐film assemblies, chemiresistive responses to binary and ternary gas mixtures were examined. Ammonia (NH_3_) was introduced as a second probe gas by bubbling N_2_ through aqueous ammonium hydroxide using a controlled flow system, generating well‐defined NH_3_/H_2_O vapor mixtures. In contrast to CO, NH_3_ introduces additional interaction pathways due to its strong Lewis basicity, enabling not only metal‐centered Type‐I adsorption at exposed nanoparticle sites but also Type‐II interactions involving proton transfer or ionic pairing with functionalized molecular linkers, along with less‐specific Type‐III partitioning within the organic matrix. Chemiresistive responses of different alkyl dithiol‐linked Au_2nm_ films toward NH_3_/H_2_O binary mixtures show positive resistance changes (Figure 4A; Figure S7), characteristic of Type‐I interactions between NH_3_ and electronically active Au sites that perturb interparticle tunneling barriers. Type‐III vapor partitioning into the ligand matrix further modulates response magnitude, particularly under humid conditions. Sensitivity exhibits a clear dependence on linker chain length, DDT‐Au_2nm_ < ODT‐Au_2nm_ < NDT‐Au_2nm_ < PDT‐Au_2nm_ (Figure S8), correlating with the relative degree of exposed Au surface sites. Films with shorter‐chain linkers display more disordered packing and greater surface accessibility, indicating that NH_3_ binding (H_3_N→Au) is governed primarily by the availability of catalytic Au sites.

Another sensing mechanistic pathway involved the binding of NH_4_^+^ to the ligand immobilized on the nanoparticles’ linking/capping shells (NH_3_ + CO_2_H → NH_4_^+^ CO_2_^−^), which is exemplified by MUA‐Au_2nm_ thin film assemblies of gold nanoparticles [28] (Figure 4B). MUA‐Au_2nm_ thin film assembly features partial ligand exchange of MUA with DT capped on Au NPs followed by interparticle linking via hydrogen bonding cross NPs, which also leaves un‐hydrogen bonded carboxylic acid groups in the MUA‐DT mixed capping layer on the NPs.

A markedly different behavior is observed for MUA‐linked Au_2nm_ nanoparticle films, as shown in Figures 4B and S9. In this case, exposure to NH_3_ results in a negative chemiresistive response, indicating a fundamentally different sensing mechanism. The carboxylic acid groups of MUA enable strong Type‐II interactions, forming ammonium–carboxylate ion pairs, which increases the effective dielectric constant of the interparticle medium and alters charge hopping probabilities, producing a resistance decrease that dominates over Type‐I metal–adsorbate effects. The polarity inversion observed for MUA‐Au_2nm_ provides direct experimental evidence that ligand functionality can be used to selectively activate Type‐II interactions and control the sign and magnitude of chemiresistive responses.

Change in dielectric properties are known to cause changes in the electrical conductivity in both positive and negative ways depending on the magnitude of the medium's dielectric constant [34]. In this case, the formation of an ionic pair of –CO_2_^−^—NH_4_^+^ in the linking/capping shells of the assembled nanoparticles is believed to be responsible for the significant change in the dielectric properties of the thin film. The contrasting response polarities and sensitivities among PDT‐, alkyl‐thiol‐, and MUA‐linked nanoparticle assemblies highlight the critical role of molecular interface design in programming sensor behavior. Importantly, the coexistence of Type‐I and II‐dominated sensing elements within a sensor array expands the dimensionality of response patterns, which is advantageous for multicomponent gas analysis. PCA score plots based on sensor array responses reveal clear separation among NH_3_, H_2_O, and mixed vapor samples, with mixture compositions distributed systematically between the single‐analyte clusters (Figure S10). This separation reflects the distinct and additive contributions of Type‐I metal adsorption, Type‐II ionic interactions, and Type‐III partitioning processes at the heterogeneous sensing interfaces. At fixed NH_3_/H_2_O ratios, the data points align along well‐defined trajectories in principal component space, indicating that the sensor array responses retain quantitative information on mixture composition.

The sensing mechanisms are further assessed by fitting the response profiles (Figure S11) for PDT‐Au_2nm_ and MUA‐Au_2nm_ films to the NH_3_/H_2_O binary mixtures. For PDT‐Au_2nm_, fitting the data in terms of NH_3_ yields *k*_a_ ∼3 × 10^−5^ s^−1^. However, when plotting against H_2_O, a higher *k*_a_ (3 × 10^−4^ s^−1^) is obtained, which is close to *k*_a_ for H_2_O only (2 × 10^−4^ s^−1^), suggesting significant adsorption of H_2_O vapor in the disordered capping/linking structures. For MUA‐Au_2nm_, fitting the negative response profiles in terms of NH_3_ yields *k* ∼ 4 × 10^−4^ s^−1^. By plotting against H_2_O, the value of *k*_a_ (7 × 10^−5^ s^−1^) is much smaller than *k*_a_ for H_2_O only (2 × 10^−4^ s^−1^). This result reflects the dominance by NH_3_ adsorption because of the coexistence of Type‐I and II‐dominated sensing elements.

**FIGURE 5:**
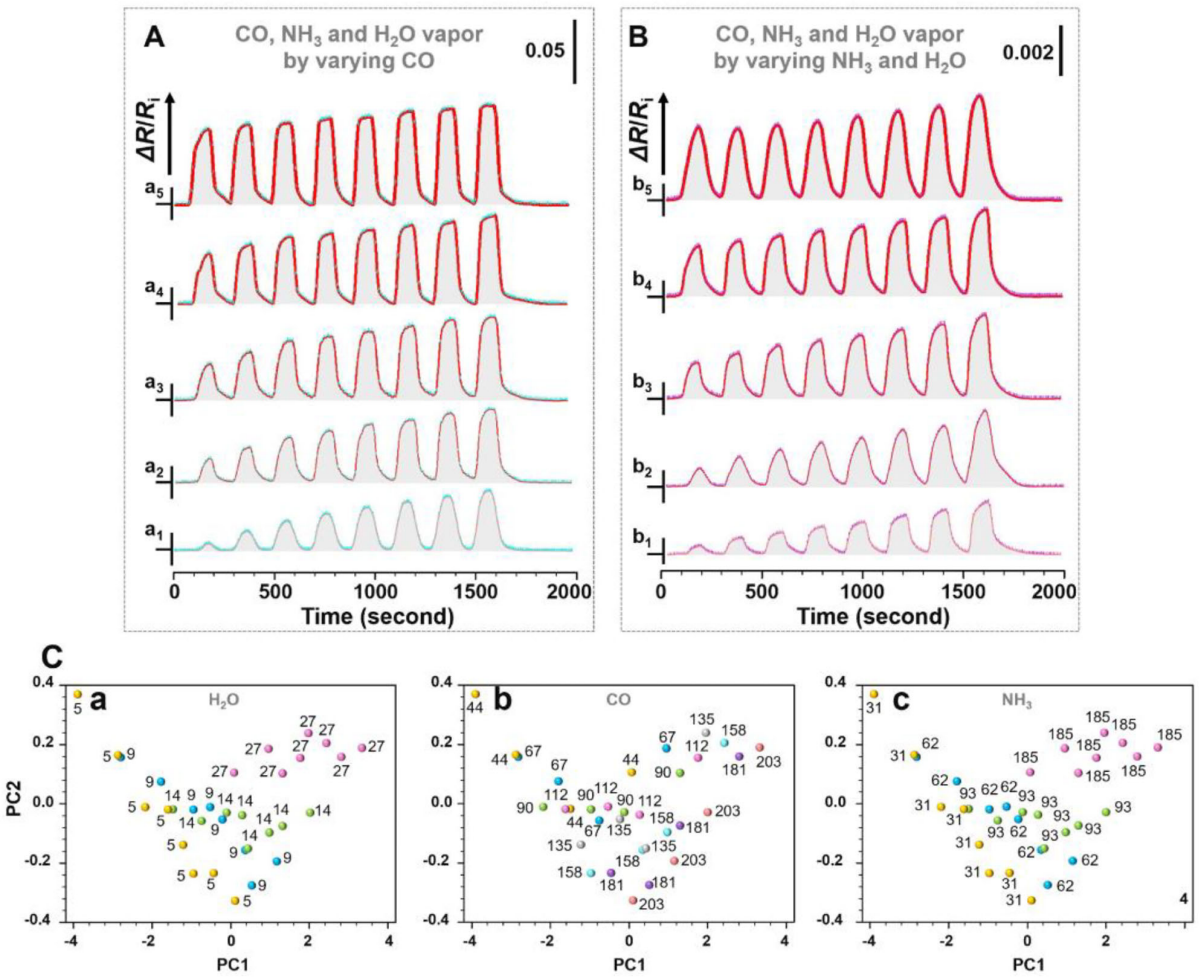
Chemiresistive responses of PDT‐Au_2nm_ to ternary mixtures. (A) Response profiles to ternary mixtures of CO, NH_3_ and H_2_O vapor by varying CO (44, 67, 90, 113, 136, 159, 182, and 205 ppm) while keeping NH_3_ and H_2_O vapor at a series of constant concentrations (NH_3_/H_2_O: a_1_: 0/0, a_2_: 31/5, a_3_: 62/9, a_4_: 93/14, and a_5_: 185/27) for PDT‐Au_2nm_ sensing films. (B) Response profiles to ternary mixtures of CO, NH_3_ and H_2_O vapor by varying NH_3_ and H_2_O vapor concentration (NH_3_/H_2_O: 31/5, 62/9, 93/14, 124/20,155/25, 186/31, 217/36, and 248/41 ppm) while keeping CO at a series of constant concentrations (b_1_: 0, b_2_: 44, b_3_: 67, b_4_: 90, b_5_: 158 ppm) for PDT‐Au_2nm_ sensing films. Differences in response magnitude relative to PDT‐Au_2nm_ films arise from linker‐dependent modulation of interparticle spacing and Type‐I site accessibility, while preserving additive interaction behavior. *Note*: see Figure S12 for response sensitivities. (C) PCA score plots for sensor array responses to ternary mixtures, which are plotted in terms of individual component: (a) H_2_O, (b) CO, and (c) NH_3_, as well as their concentrations (ppm). Distinct clustering and systematic trajectories reflect orthogonal contributions from Type‐I metal adsorption, Type‐II ionic interactions, and Type‐III vapor partitioning encoded by the sensing interfaces. *Note*: The comparative data for ODT‐Au_5nm_ sensing films are shown in Figures S14 and S15.

These results establish that the nanoparticle assemblies provide a versatile platform for constructing sensor arrays capable of resolving multicomponent gases through rational interface design. We next examined ternary mixtures of CO, NH_3_, and H_2_O to evaluate whether these different interaction pathways remain addressable under increased chemical complexity, which represents a stringent test of the molecularly programmed sensing interfaces, as multiple analytes compete for metal sites, functional groups, and the ligand matrix. Figures 5A and S12a present the chemiresistive responses of PDT‐Au_2nm_ and ODT‐Au_5nm_ sensing films to CO, NH_3_, and their binary and ternary mixtures under controlled concentrations. When two components are held constant, the response varies linearly with the concentration of the third analyte, indicating additive contributions to the overall signal. The results demonstrate relatively good linear relationships with CO concentration at each NH_3_‐H_2_O concentration for both PDT‐Au_2nm_ and ODT‐Au_5nm_ films. These trends suggest that CO adsorption at exposed metal sites (Type‐I), NH_3_ interactions with both metal sites and functional groups (Type‐I/II), and H_2_O partitioning within the ligand matrix (Type‐III) operate largely independently. Similar linear scaling behavior is observed for these two sensing films being tested in a different way of varying the relative concentrations (Figure 5B; Figure S12b). Although the overall response magnitudes differ due to these variations, the persistence of linear response manifolds across different linker systems demonstrates that the additive nature of Type‐I, II, and III interactions.

The mechanisms were also assessed by fitting response profiles to the ternary mixtures (see Figure S13). For PDT‐Au_2nm_ film, increasing CO concentration of CO in the absence (a_1_) and the presence of NH_3_/H_2_O (a_2_ to a_5_) caused a subtle increase in *k*_a_ (2 × 10^−4^ s^−1^ (a_1_) to 3 × 10^−4^ (a_3_) and 6 × 10^−4^ s^−1^ (a_4_)). Increasing NH_3_/H_2_O in the absence (b_1_) and the presence of CO (b_2_ to b_5_) yielded 6 × 10^−4^ (b_1_), 6 × 10^−4^ (b_3_), and 9 × 10^−4^ s^−1^ (b_4_) for NH_3_ and ∼ 4 × 10^−3^ s^−1^ (b_1_), 4 × 10^−3^ (b_3_), and 6 × 10^−3^ s^−1^ (b_4_) for H_2_O. For ODT‐Au_5nm_, featuring a longer chain linking structure, this film showed similar characteristics but different magnitudes (Figures S14 and S15). *k*_a_ remained largely unchanged for CO (e.g., a_1_, 3 × 10^−4^ s^−1^; a_3_ and a_4_: 1 × 10^−3^ s^−1^) in the absence and the presence of NH_3_/H_2_O. While *k*_a_ versus NH_3_ is largely unchanged (e.g., b_1_, b_3_, b_4_: 4 × 10^−4^ to 4 × 10^−4^ to 3 × 10^−4^ s^−1^) for NH_3_/H_2_O in the absence and presence of CO, *k*_a_ values for H_2_O were higher but also stable (e.g., b_1_, b_3_, b_4_: 3 × 10^−3^ to 2 × 10^−3^ to 2 × 10^−3^ s^−1^). These differences stem from variations in capping/linking molecular packing, where the longer‐chain ODT linker creates fewer Type‐I and more Type‐III sites compared to PDT.

Quantitatively, limits of detection (LOD) were also estimated from the sensitivity (*s*, response vs. concentration slope) and the standard deviation of the blank's noise (i.e., 3σ/*s*). For the Au NP‐based sensing thin films (σ ∼ 2 × 10^−6^ ∼1 × 10^−3^, and *s* ∼ 1 × 10^−6^ ∼5 × 10^−4^, see SI), LODs were found to fall in the range of 0.3–8 ppm for CO (with or without H_2_O) and 0.2–20 ppm for NH_3_ (with H_2_O), depending on a combination of alkyl length and functional group of the capping/linking molecules in the thin film assemblies.

To visualize and quantify array‐level discrimination of ternary gas compositions, PCA score plots (Figure 5C; Figure S16) reveal distinct clustering of CO, NH_3_, H_2_O, and their ternary mixtures, with systematic trajectories corresponding to changes in mixture composition. These separations reflect the chemically orthogonal response contributions encoded by different interaction types. Qualitatively, variations along the principal‐component axes are consistent with differing contributions from metal‐site adsorption (Type‐I), ligand‐mediated interactions (Type‐II), and matrix partitioning processes (Type‐III). Because PCA identifies statistical variance rather than mechanistic causality, these assignments should be viewed as interpretive correlations rather than direct proof of individual interaction mechanisms. The coexistence of Type‐I metal–NH_3_ coordination and Type‐II acid–base interactions with linker functionalities explains both the enhanced sensitivity and polarity modulation observed, in agreement with the interaction hierarchy depicted in Scheme 1.

### 2.3 Switchable Response Mechanisms

**FIGURE 6:**
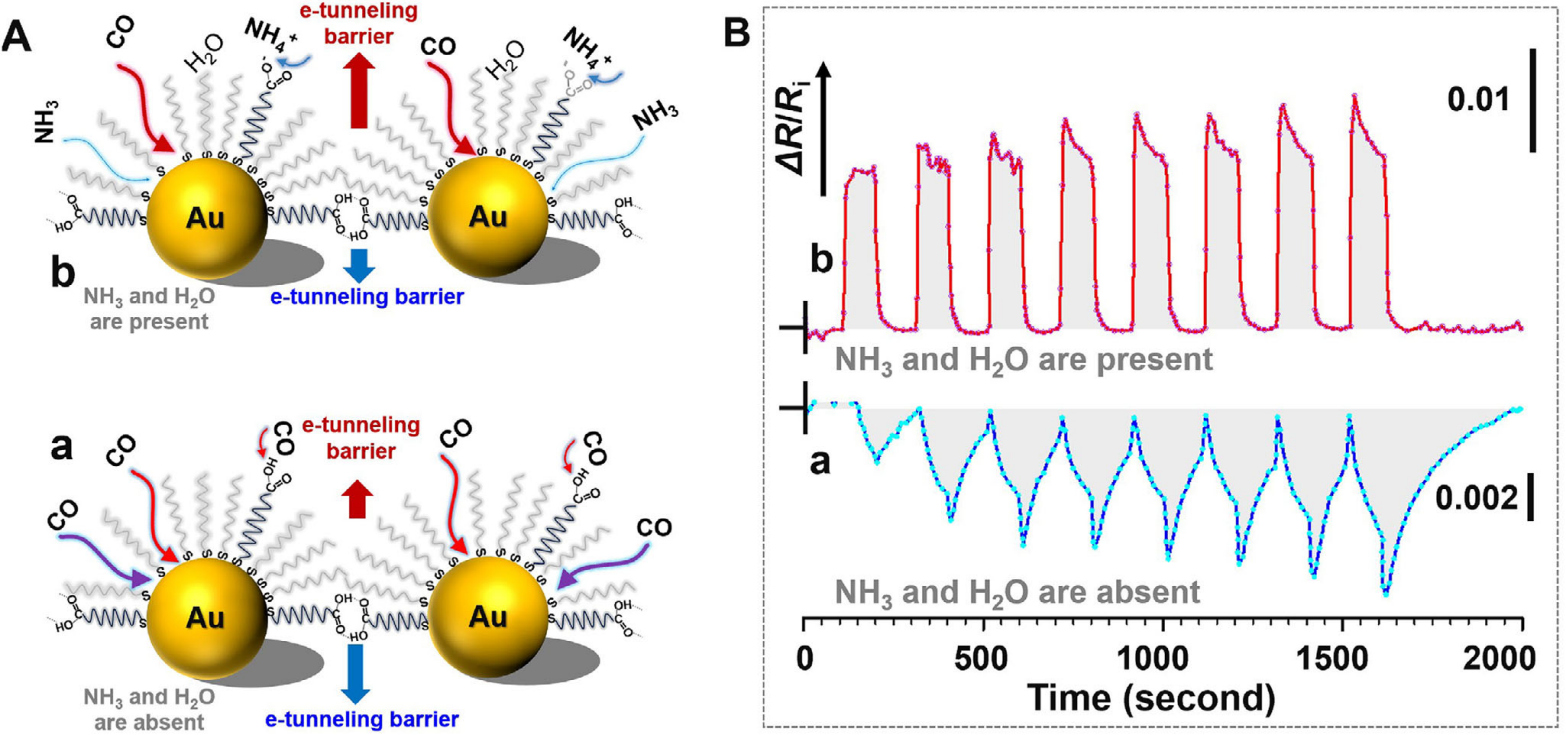
Demonstration of switching of response mechanisms for MUA‐Au_2nm_ under different conditions. (A) Schematic illustration of the interfacial binding sites and mechanism. (B) Response profiles to CO (44, 67, 89, 112, 135, 158, 180, and 203 ppm) in the absence (a) and presence (b) of NH_3_/H_2_O (93/14 ppm). See Figure S17 for plots in terms of the response sensitivities. The response polarity inversion observed for MUA‐Au_2nm_ highlights the dominance of Type‐II ionic interactions over Type‐I metal‐site adsorption, demonstrating ligand‐controlled competition between sensing pathways. Note that capping and linking molecules are shown on a portion of the nanoparticles and are not to scale, depicting defects in an idealized monolayer packing on the surface.

Finally, we demonstrate the viability of sensing mechanism switching depending on the type of interparticle linking and specific interaction of the gas molecules with the film. This is illustrated by MUA‐Au_2nm_ as an example (Figure 6). MUA‐Au_2nm_ films exhibit negative response profiles upon exposure to CO only (a). In contrast, the film shows positive response profiles to CO in the presence of NH_3_ + H_2_O (b). In case‐a, Type‐I interaction is dominant, but the thin film assembly features a large interparticle gap defined by head‐head hydrogen bonding of two MUA linkers. In case‐b, there is a strong Type‐II interaction between NH_3_ and carboxylate groups, which increase the effective dielectric constant of the interparticle medium and outweigh metal‐site adsorption effects. The contrasting behaviors of these two systems provide direct experimental evidence for the coexistence and competition of multiple interaction pathways within a single sensing platform.

While the strong Type‐II interaction leads to the formation of ammonium–carboxylate ion pairs in MUA‐Au_2nm_ film in the presence of H_2_O, which increases the effective dielectric constant of the interparticle medium and alters charge hopping probabilities and should produce a resistance decrease, the CO adsorption apparently put Type‐I metal–adsorbate back to dominance, leading to positive responses (b in Figure 6A,B). The presence of the negative response effect associated with Type‐II can still be seen by the initial positive followed by negative response profiles. Interestingly, in the absence of NH_3_ and H_2_O, MUA‐Au_2nm_ shows small‐magnitude negative responses to CO (a in Figure 6A,B). In this case, the head‐head hydrogen bonding defines a large interparticle gap in MUA‐Au_2nm_, which combines with the dielectric effect to make the overall resistance decrease with CO adsorption. Nevertheless, subtle CO‐metal led resistance increase can still be observed by the initial negative response profile followed by positive response shifting.

A plausible interpretation of the observed response switching involves changes in the relative contributions of interparticle tunneling, thermally activated hopping, and local dielectric modulation arising from variations in linker structure and analyte adsorption. Changes in interparticle spacing, surface dipoles, and molecular adsorption may alter the effective tunneling barriers and charge‐transport pathways within the nanoparticle assemblies. While the present experiments do not directly resolve the microscopic transport mechanism, the observed response polarity changes are consistent with analyte‐induced modulation of interparticle electronic coupling and dielectric environment. Future studies combining electrical measurements with spectroscopic and theoretical analyses may further elucidate the detailed transport processes responsible for the switching behavior. In n‐type assemblies, electron donation from NH_3_ lowers interparticle tunneling barriers, leading to an exponential increase in tunneling probability and a corresponding decrease in resistance. Together, these mechanisms highlight the exceptional tunability of molecularly assembled films, where positioning the system near a transport transition enables highly sensitive chemiresistive responses to subtle structural or chemical perturbations.

These results establish generalizable design principles for chemiresistive sensing using molecularly mediated nanoparticle assemblies. Selective detection and mixture resolution emerge from the deliberate programming of interfacial interaction pathways, where metal‐site adsorption (Type‐I), ligand‐mediated specific or ionic interactions (Type‐II), and nonspecific vapor partitioning within the organic matrix (Type‐III) can be independently tuned through nanoparticle composition, linker chemistry, and film architecture. The orthogonal and additive nature of these interaction modes enables predictable scaling of sensor responses from single analytes to binary and ternary mixtures while preserving chemically specific signatures under increasing environmental complexity. In addition, across all analytes and mixtures examined, the chemiresistive responses were fully reversible upon purging with carrier gas, with no detectable baseline drift over repeated exposure cycles, indicating stable interfacial interactions within the measurement timeframe. These findings demonstrate that rational interface engineering offers a mechanistically grounded strategy for constructing multifunctional chemiresistive sensor arrays capable of addressing real‐world gas sensing challenges. The additive yet distinguishable contributions of Type‐I, II, and III interactions enable predictable scaling from single analytes to ternary mixtures, validating the design rules summarized schematically in Scheme 1.

## 3 Conclusion

In summary, this work establishes a mechanistically guided framework for understanding and programming chemiresistive responses in molecularly assembled nanoparticle films through three cooperative interaction pathways: metal‐site adsorption (Type‐I), ligand‐mediated interactions (Type‐II), and matrix partitioning effects (Type‐III). Using representative analytes and their binary and ternary mixtures, we demonstrate how molecular interface design can selectively activate and combine these interaction modes to generate chemically distinguishable sensing responses. These findings provide design principles for molecularly assembled nanoparticle chemiresistors and offer a foundation for future development of multifunctional sensing arrays operating in chemically complex environments. Furthermore, the integration of artificial intelligence‐enhanced optimization methods, such as multimodal machine learning and intelligent genetic algorithms, represents a critical step forward in refining these arrays for advanced wireless environmental monitoring and healthcare diagnostics [35].

## 4 Experimental Section

### 4.1 Chemicals and Gas Generation

Carbon monoxide (CO) streams with variable concentrations were generated using a custom testing station equipped with digital mass flow and mixing controllers, utilizing a primary gas source of 1 vol % CO balanced in N_2_. Online sample analysis was performed using a gas chromatograph (Shimadzu GC‐8A) equipped with a thermal conductivity detector (TCD). Ammonia (NH_3_) gas was generated by bubbling N_2_ carrier gas through an aqueous ammonium hydroxide (NH_4_OH) solution. The resulting NH_3_ concentration was precisely controlled by adjusting the solution temperature, pH, N_2_ flow rate, and NH_4_OH concentration. To ensure a dry gas stream, water vapor was selectively removed using a cold trap or an ammonia‐inert chemical desiccant. This configuration reliably delivered stable NH_3_ concentrations ranging from 50 to 1500 ppm.

### 4.2 Synthesis of Metal Nanoparticles

Gold and copper nanoparticles (2–5 nm) were synthesized using modified two‐phase and single‐phase protocols, with particle size precisely controlled via thermally activated processing [29, 30]. For the gold nanoparticles (average size ∼2 nm, Figure S18), a standard two‐phase method was employed to encapsulate the metallic cores within decanethiolate (DT) monolayer shells (denoted as Au_2nm_‐DT) [29]. Briefly, aqueous tetrachloroaurate (AuCl_4_^−^) was phase‐transferred into toluene using tetraoctylammonium bromide (TOABr) as the phase‐transfer agent, followed by the addition of DT. Subsequent reduction using sodium borohydride (NaBH_4_) yielded the DT‐capped nanoparticles. Copper nanoparticles (average size ∼5 nm, Figure S19) functionalized with oleylamine (OAM) and oleic acid (OAC) ligands (OAM/OAC‐Cu_5nm_) were synthesized according to a previously reported method [36]. Copper(II) acetylacetonate (Cu(acac)_2_) was reduced by 1,2‐hexadecanediol in dioctyl ether, utilizing OAC and OAM as co‐capping agents. The reaction temperature was precisely maintained between 150 and 210°C to achieve the target size. These protocols consistently yielded a size monodispersity within ±5%–10%. The size, morphology, and structural characteristics of the resulting nanoparticles were evaluated via transmission electron microscopy (TEM) and Fourier‐transform infrared spectroscopy (FTIR).

### 4.3 Fabrication of Interdigitated Microelectrode (IME) Devices

Interdigitated microelectrode (IME) devices were fabricated at the Cornell NanoScale Science & Technology Facility (CNF) using standard photolithographic protocols. The IME design featured 150–300 pairs of gold (Au) electrodes patterned on both rigid glass and flexible polyethylene terephthalate (PET) substrates. To ensure robust metal adhesion, a 20 nm titanium (Ti) adhesion layer was deposited via vacuum sputtering, followed by the deposition of a 130 nm Au film (achieving a total electrode thickness of 150 nm). The electrode geometries were defined by a finger width (FW) and finger spacing (FS) of 5–10 µm, and a finger length (FL) of 100–200 µm. These IMEs incorporated a plug‐in architecture to allow seamless integration into compact array sockets equipped with miniaturized gas compartments. These devices, characterized by precise finger pair (FP), FS, FW, and FL parameters, served as the structural foundation for the subsequent deposition of nanostructured thin films and sensing arrays.

### 4.4 Preparation of Nanoparticle Thin‐Film Assemblies and Sensor Arrays

Thin‐film assemblies (50–500 nm thick) comprising metal nanoparticles (e.g., Au) capped and crosslinked by alkyl thiols, dithiols, or dicarboxylic acids were prepared via a one‐step exchange‐crosslinking precipitation route [29]. The interparticle spatial properties are governed by Au–S covalent bonding, hydrogen bonding, and hydrophobic/hydrophilic interactions. By varying the length of the methylene spacers within the linker network—structurally represented as (RS‐)_n‐n'_ Au_m_ (‐S‐(CH_2_)_n_‐S‐)Au_m_ (‐SR)_n‐n'_ and (RS‐)_n‐n'_ Au_m_ (‐SR'CO_2_H)_n_···_n'_(HO_2_C R'S‐)Au_m_ (‐SR)_n‐n'_ (where R represents an alkyl group)—the edge‐to‐edge interparticle distance was tuned between 0.5 and 2.5 nm. These interactions dictate network assembly through covalent thiolate bonding, carboxylic acid dimerization via hydrogen bonding, and hydrophobic interdigitation of alkyl capping groups (Figure S20). The evaluated linkers included 11‐mercaptoundecanoic acid (MUA), 1,9‐nonanedithiol (NDT), 1,12‐dodecanedithiol (DDT), 1,8‐octanedithiol (ODT), and 1,5‐pentanedithiol (PDT).

For film deposition, the IMEs were cleaned with piranha solution (H_2_SO_4_:H_2_O_2_ 3:1 v/v), sonicated in deionized (DI) water, and rinsed with ethanol. The pre‐cleaned substrates were immersed vertically into the assembly solution for controlled durations, rinsed with hexane, and dried under a continuous N_2_ stream. Specific combinations of varied IME parameters and MUA‐mediated Au_2nm_ nanoparticle assemblies are detailed in Figure S21 and Table S3. Similarly, OAM/OAC‐capped Cu nanoparticles were assembled using 1,6‐hexanediamine (HDA) or 1,8‐octanediamine (ODA) linkers.

### 4.5 Plasma Treatment

Organic capping ligands were removed from the gold nanoparticles via reactive oxygen (O_2_) plasma treatment using a plasma cleaner (Harrick Plasma, PDC‐32G) equipped with a radiofrequency (RF) power source. This process oxidatively converted the organic ligands into volatile gases (e.g., CO_2_, H_2_O), which were evacuated under vacuum to expose pristine NP surfaces and generate active sites for catalysis and gas sensing. By precisely maintaining the RF power at 6.8 W (low power setting) and varying the exposure duration between 0.2 and 5 min, the organic monolayer was thoroughly eliminated without inducing NP sintering, aggregation, or sub‐surface oxidative damage.

### 4.6 FTIR Characterization

The structural and compositional properties of the nanoparticle thin‐film assemblies were evaluated via infrared reflection absorption spectroscopy (IRRAS) using a Fourier‐transform infrared (FTIR) spectrometer (Nicolet 760 ESP) equipped with a liquid nitrogen‐cooled mercury cadmium telluride (HgCdTe) detector. Spectra were acquired in external reflection mode using an incident angle of 82° with *p*‐polarized light at a spectral resolution of 2 cm^−1^. To conduct accurate spectral and background analysis, a gold‐coated slide modified with a self‐assembled monolayer of deuterated octadecanethiolate (ODT‐*d*_37_) served as the reference substrate.

### 4.7 Evaluation of Sensor Responses

The vapor‐sensing performance of the nanostructured thin films was evaluated using an automated array testing system integrated with a multi‐channel resistance meter, a frequency counter, and a digital flow‐controlled vapor generation and mixing network. This system featured a low dead‐volume manifold utilizing Swagelok Modular Platform Components (MPC) technology to precisely switch gaseous analytes and establish comprehensive sensor profiles. Analyte concentrations for single and mixed vapor streams were independently verified using an on‐line gas chromatograph (Varian CP‐3800) equipped with a flame ionization detector (FID). All gas concentrations are reported as molar ppm, which can be converted to volume ppm using a multiplication factor of 24.5 under standard ambient conditions.

Electrical resistance was measured by housing the flexible sensor devices within a custom‐built, low dead‐volume manifold (200 mL) interfaced with the vapor distribution network. A computer‐controlled multi‐channel data acquisition system (Keithley, Model 2700) continuously monitored the film resistance. The sensor response is expressed as the relative change in electrical resistance, defined by the equation Δ*R*/*R* = (*R*_gas_—*R*_0_)/*R*_0_, where *R*_gas_ and *R*_0_ represent the resistance in the presence of the analyte gas and the baseline carrier gas, respectively, following standard chemiresistive evaluation protocols [28, 29, 37]. All sensing experiments were performed at room temperature (25°C). While operating under ambient air or complex atmospheres represents a critical direction for practical implementations, high‐purity N_2_ was intentionally utilized as the carrier gas in this work. This choice isolated and systematically elucidated the intrinsic interfacial interactions of CO, NH_3_, and H_2_O by eliminating confounding variables introduced by ambient oxygen adsorption or competing surface oxidation pathways.

## Conflicts of Interest

The authors declare no conflicts of interest.

## Supporting Information

Supporting Information is available. Details related to some experimental and analysis sections including response profiles, response sensitivities, fitting to response kinetics, principal component analysis (PCA) score plot, schematic illustration of the thermally activated evolution process, and TEM images.

## Supporting information




**Supporting File**: smll74676‐sup‐0001‐SuppMat.pdf.

## Data Availability

The data that support the findings of this study are available from the corresponding author upon reasonable request.
