# Supplementary material for: Molecularly Mediated Assembly of Metal Nanoparticles With Multifunctional Interfaces for Selective Detection of Small Gas Molecules
Source: Small. 2026 Jul 23;22(52):e74676. doi: 10.1002/smll.74676 (PMC13580240; doi:10.1002/smll.74676)
Supplement: Supplementary file 1 [file SMLL-22-e74676-s001.pdf]

Supporting Information

Molecularly Mediated Assembly of Metal Nanoparticles with Multifunctional Interfaces for Selective Detection of Small Gas Molecules

*Han-Wen Cheng, Jin Luo, Xiajing Shi, Guojun Shang, Dong Dinh, Lidia Gebre, Zeqi Li, Seyed Danial Mousavi, Zakiya Skeete, Ke Pei, Wenbin You, Susan Lu, Tony T. Yuan, and Chuan-Jian Zhong***

Table of Contents:

Supporting Tables……………………………………………………........S2-S3

Supporting Figures…………………………………………………......S4-S14

References………………………………………………………………S15

Supporting Tables:

Table S1. Prior studies establishing the structural, transport, sensing, and analytical foundations of molecularly mediated nanoparticle assemblies relevant to the present work.

Category	Key Prior Findings	Representative References
Nanoparticle synthesis	Size-controlled Au and Cu nanoparticles, and their characterizations using TEM, FTIR, XPS, etc.	[1-4]
Ligand exchange chemistry	Quantitative characterization of linker exchange and interparticle linking	[5-7]
Thin-film assembly	Ligand-exchange-crosslinking-precipitation route for nanoparticle thin film formation, morphology, and thickness evolution	[3, 8, 9]
Interparticle spacing	Linker chain length and structure dependent interparticle spacing control and characterization	[10-12]
Electrical transport	Quantitative analysis and characterization of electrical conductivity vs linker length and spacing, and thermally activated conduction mechanism	[10, 11, 13]
Device architecture	Design, fabrication, and characterization of interdigitated microelectrode (IME) devices as well as optimization	[14, 15]
Sensing mechanisms	Detection of VOCs and mixed VOCs, chemiresistive responses; sensitivity, selectivity, and LOD, sensing mechanisms	[10, 16, 17]
Sensor Arrays and Pattern Recognition	PCA/Fuzzy ARTMAP, pattern recognition, and optimization of sensor arrays	[17-20]
Stability and flexibility	Spectroscopic characterizations of the nanoparticle thin films (FTIR, UV-Vis, XPS, etc.), and mechanical and operational stability	[4, 13, 21-23]
Recent extensions	Breath VOC sensing, optimization of sensor arrays, 3D assemblies, LOD, applying AI/ML for sensor optimization and data analytics	[20, 24, 25]

Note: The present manuscript focuses on establishing a unified mechanistic framework based on Type-I, Type-II, and Type-III interactions in molecularly assembled nanoparticle chemiresistors. Because the platform has been developed over more than two decades, Table S1 summarizes representative prior studies that established nanoparticle synthesis, assembly, transport, sensing, and pattern-recognition characteristics relevant to the present work.

Table S2. Summary of classification (Total recognition rate: 85%).

Tested analytes	CO	H_2O	H_2O+CO
Sample classification #	15	15	30
Correct classification #	15	10	26
Recognition rate	1	0.667	0.867

Table S3. Design of the sensor array.

	Sensor 1	Sensor 2	Sensor 3	Sensor 4
Sensing Film	MUA-Au_{2nm}	MUA-Au_{2nm}	MUA-Au_{2nm}	MUA-Au_{2nm}
Finger Space	10 μm	10 μm	5 μm	5 μm
Finger width	10 μm	5 μm	10 μm	5 μm
Finger Length	100 μm	200 μm	200μm	100 μm

Supporting Figures:

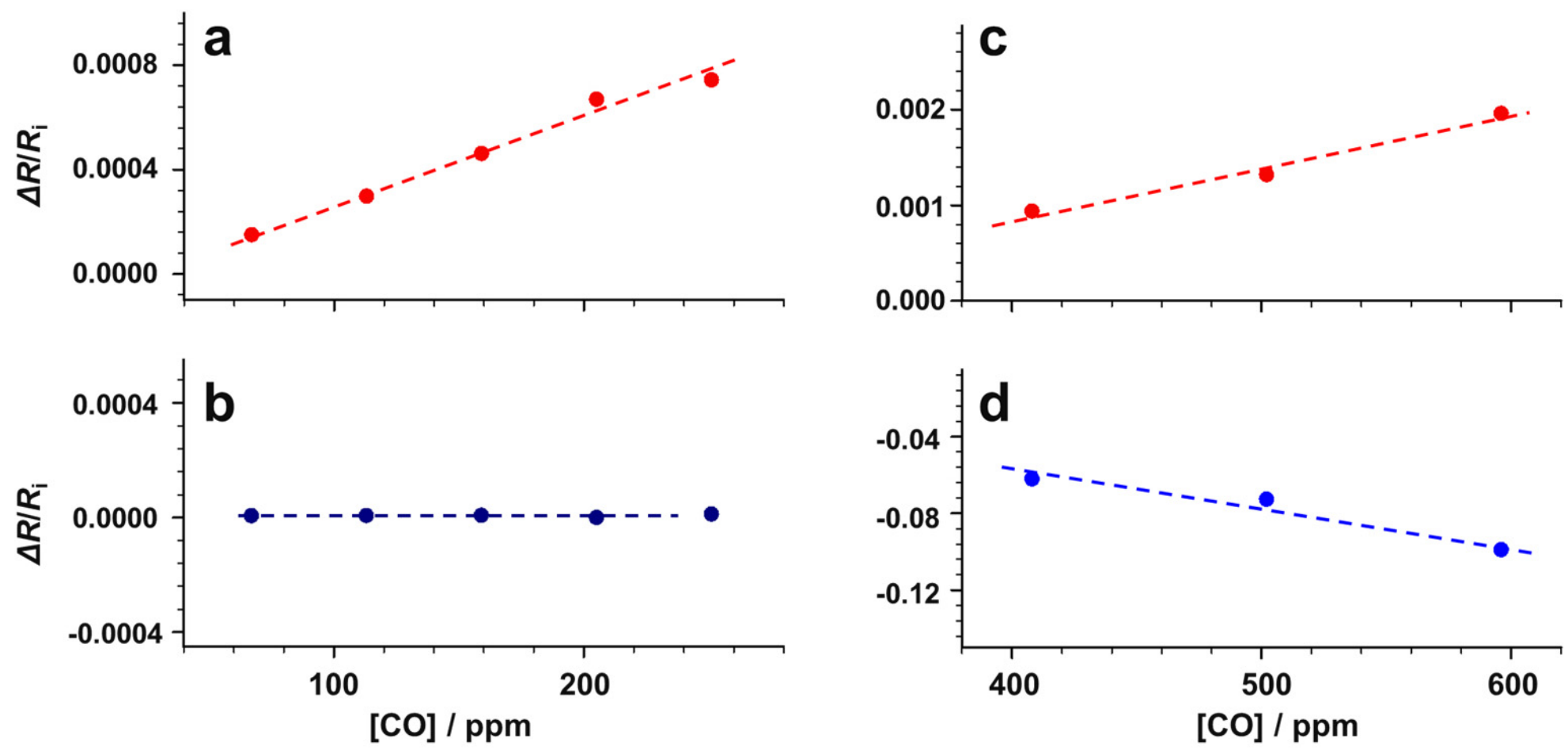

Figure S1. CO response sensitivities, corresponding to Figure 1 d-e and f-g plots. (a-b) 3.4×10^{-6} ppm^{-1} (a, LOD ~4.4 ppm (i.e., $3\times5.02\times10^{-6}/3.4\times10^{-6}$)); 3.0×10^{-8} ppm^{-1} (b). (c-d) 5.4×10^{-6} ppm^{-1} (c, LOD ~ 5.2 ppm); -2.0×10^{-4} ppm^{-1} (d).

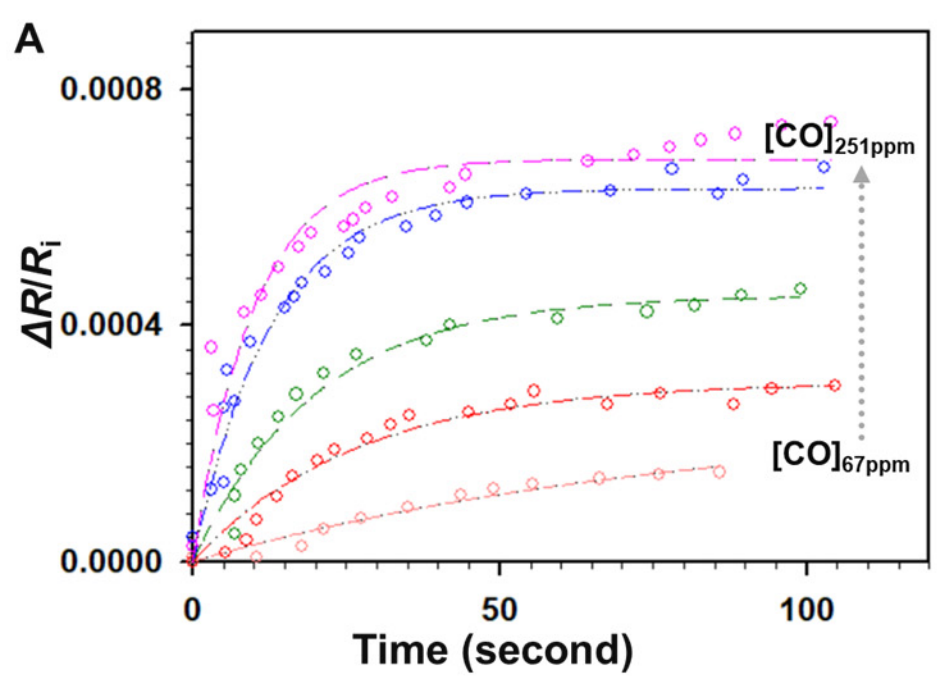

Figure S2A (corresponding to the response kinetics shown in Figure 1d)	[CO]/ppm	Fitting parameters *a*	*b*
	67	0.0002	0.0124
	113	0.0003	0.0376
	159	0.0005	0.0498
	205	0.0006	0.0790
	251	0.0007	0.0996

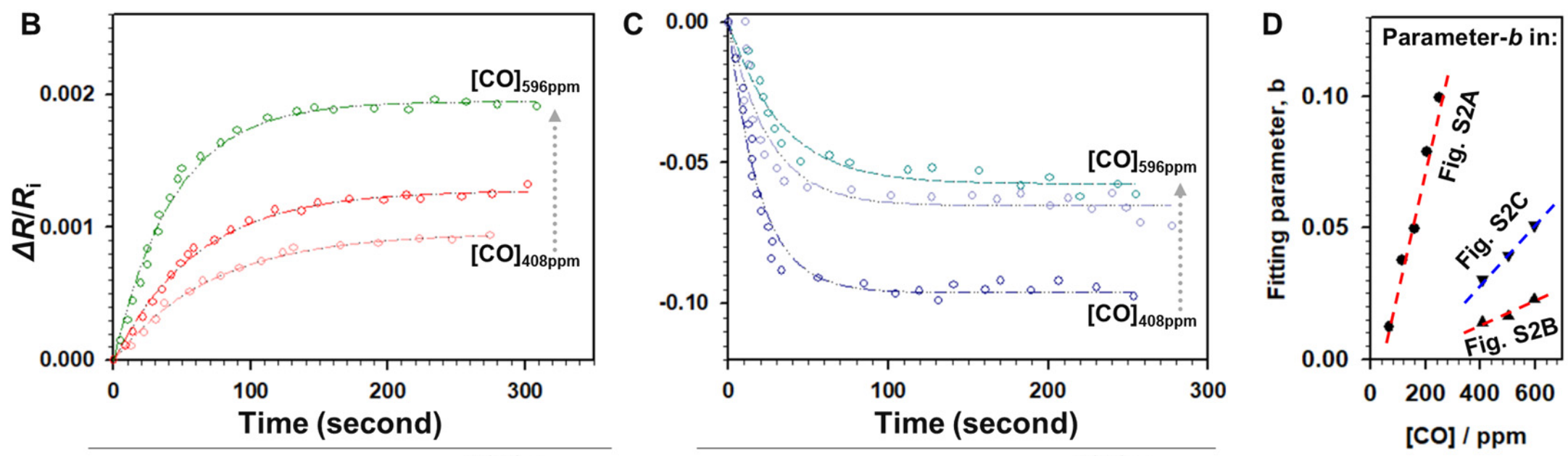

Figure S2B (corresponding to the response kinetics shown in Figure 1f)	[CO]/ppm	Fitting parameters *a*	*b*
	408	0.0010	0.0141
	502	0.0013	0.0164
	596	0.0019	0.0228

Figure S2C (corresponding to the response kinetics shown in Figure 1g)	[CO]/ppm	Fitting parameters *a*	*b*
	408	-0.0574	0.0304
	502	-0.0638	0.0396
	596	-0.0961	0.0510

Figure S2. (A, B, C) Fitting to response kinetics in Figure 1d, 1f, and 1g, respectively. Dashed lines: the theoretical fit based on Langmuir adsorption isotherm $\theta = a[1-\exp(-bt)]$, where $a = C_M/(C_M + K^{-1})$, $K=k_a/k_b$, and $b= k_aC + k_d$, where k_a is the adsorption rate constant, k_d is the desorption rate constant, and C is the CO gas concentration [26]. (D) Plots of values of b vs. CO concentration (ppm) for A, B and C. Linear regression slope, i.e., k_a: (A) 5×10^{-4} s^{-1}, (B) 5×10^{-5} s^{-1}, and (C) 1×10^{-4} s^{-1}.

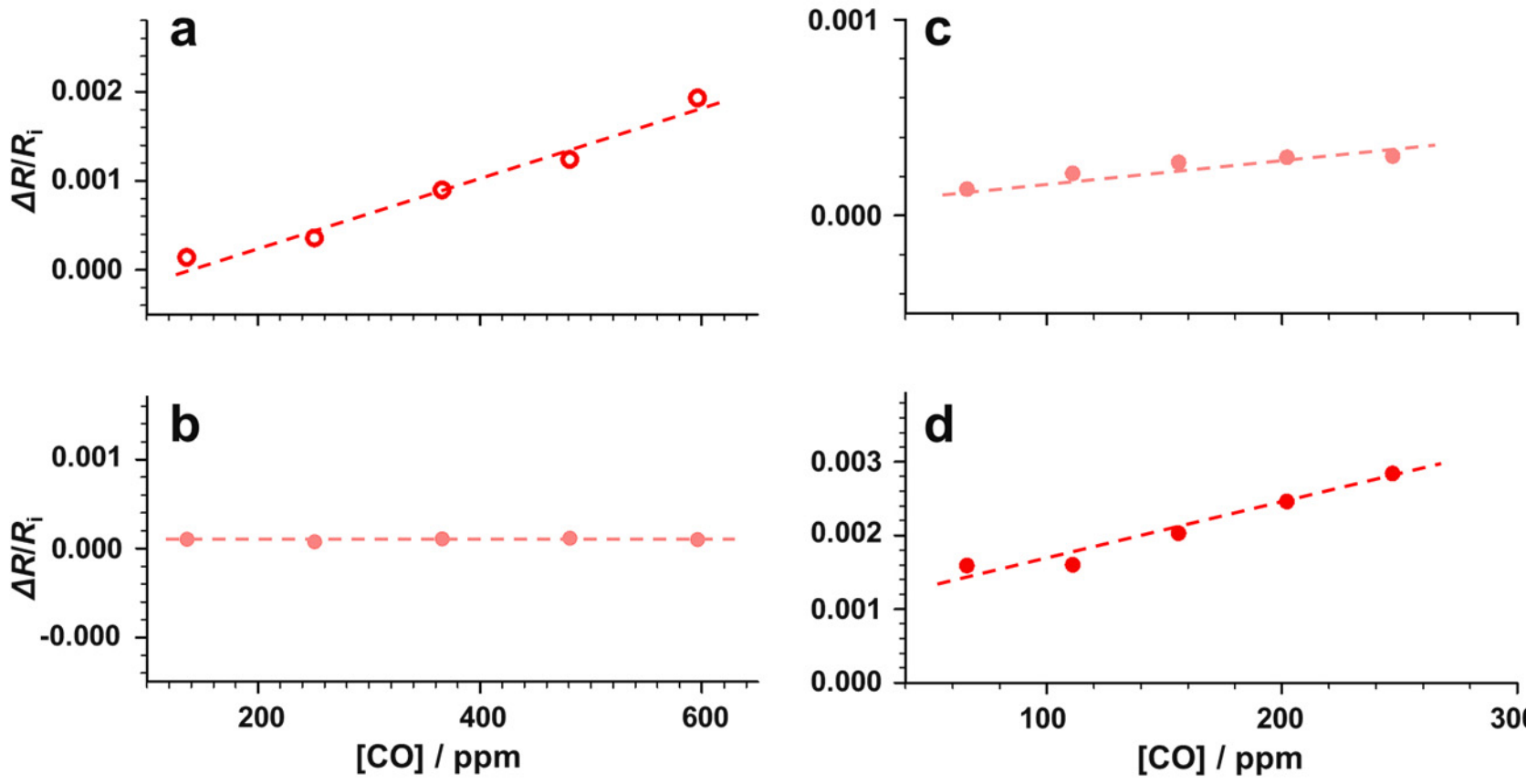

Figure S3. CO response sensitivities, corresponding to Figure 2A (a-b) and 2B (b-c) plots. (a-b) 3.9×10^{-6} ppm^{-1} (a, LOD ~9.8 ppm); 2.5×10^{-8} ppm^{-1} (b). (c-d) 9.2×10^{-7} ppm^{-1} (c, LOD ~7.5 ppm); 7.4×10^{-6} ppm^{-1} (d, LOD of ~4.7 ppm).

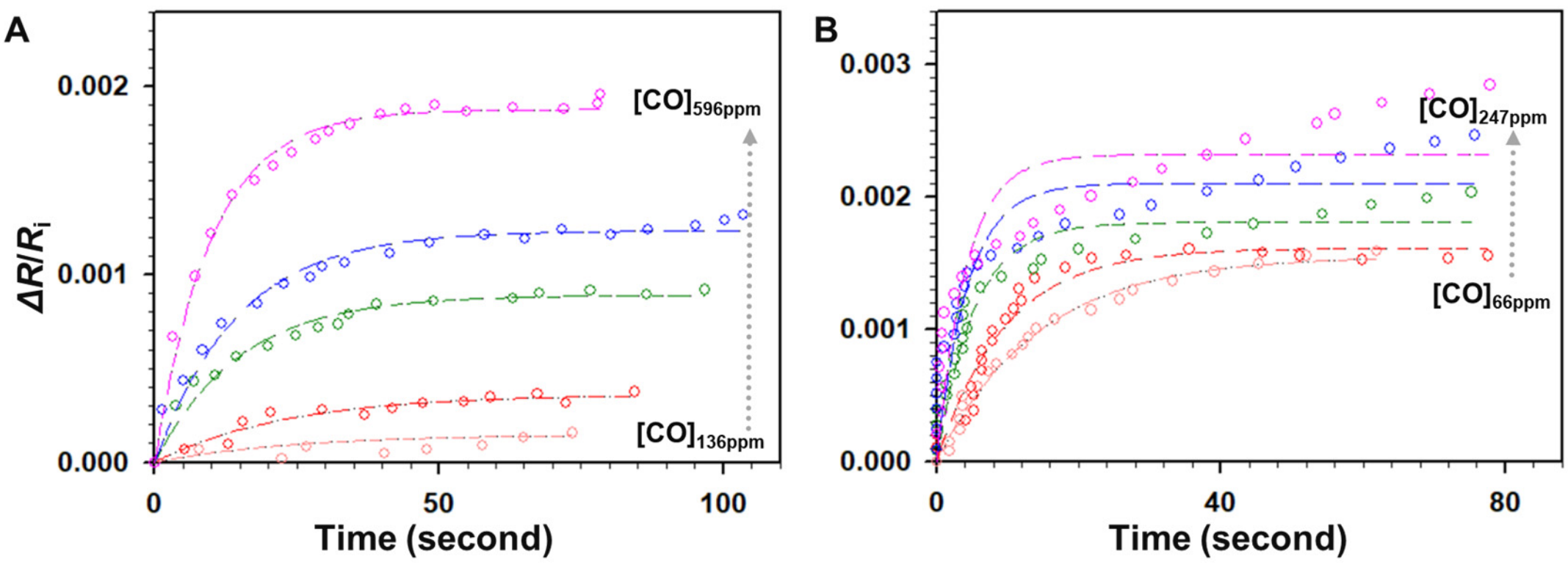

	[CO]/ppm	Fitting parameters	
		a	*b*
Figure S4A (corresponding to the response kinetics shown in Figure 2A(a))	136	0.0001	0.0410
	251	0.0004	0.0437
	366	0.0009	0.0677
	481	0.0012	0.0687
	596	0.0019	0.1008

	[CO]/ppm	Fitting parameters	
		a	*b*
Figure S4B (corresponding to the response kinetics shown in Figure 2B(c))	66	0.0015	0.0699
	111	0.0016	0.1036
	156	0.0018	0.1898
	202	0.0021	0.2280
	247	0.0023	0.2409

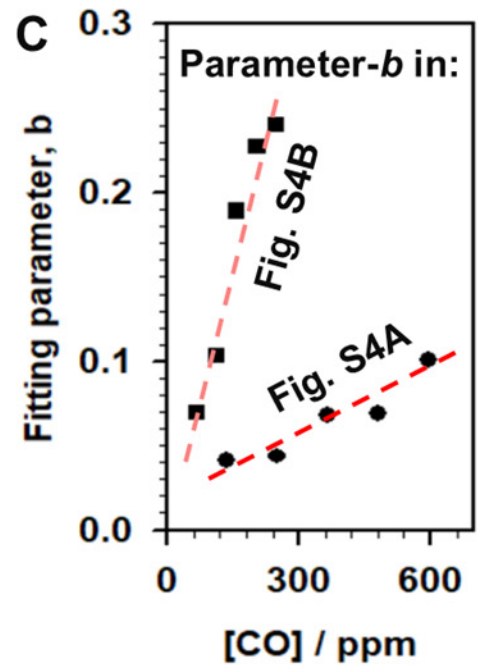

Figure S4. (A, B) Fitting to response kinetics in Figure 2A(a), and 2B(c), respectively. Dashed lines: the theoretical fit based on Langmuir adsorption isotherm. (C) Plots of values of *b* vs. CO concentration (ppm) for A and B. Linear regression slope, i.e., k_a: (A) 1×10^{-4} s^{-1} and (B) 1×10^{-3} s^{-1}.

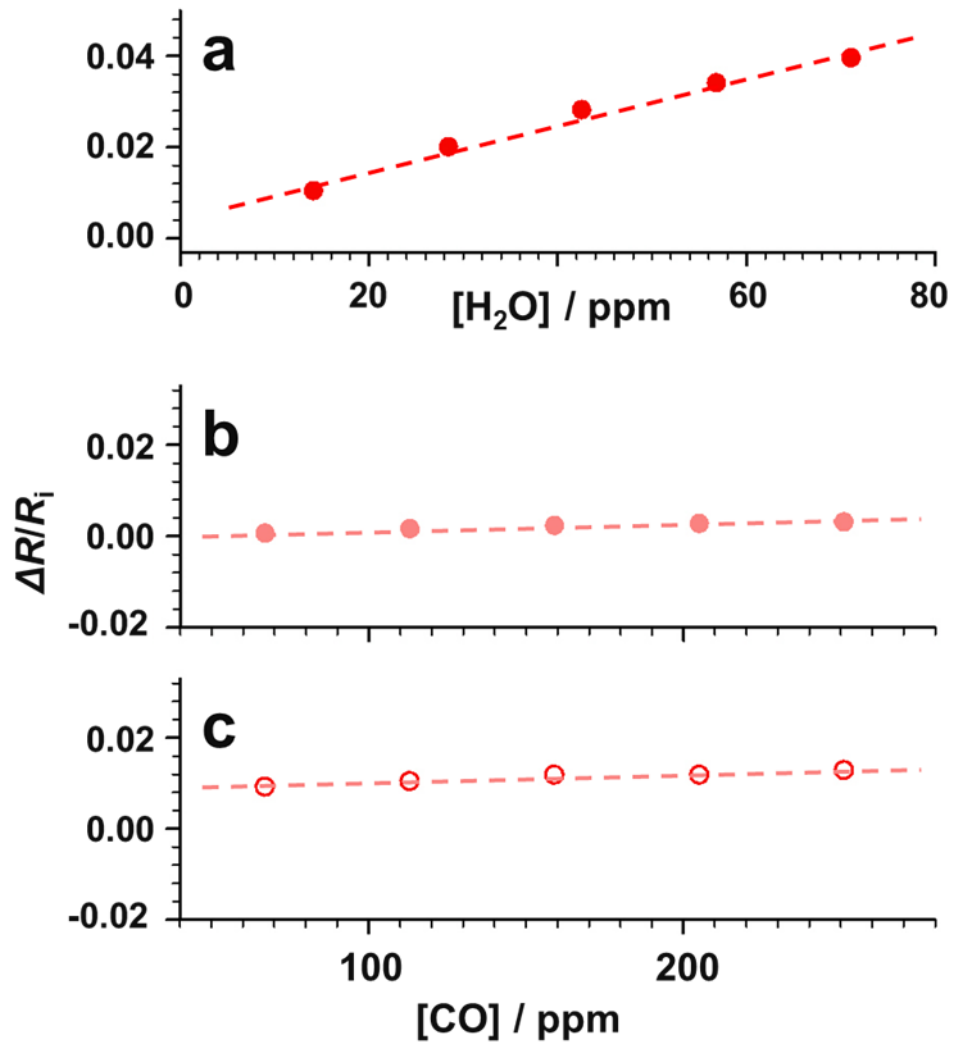

Figure S5. Response sensitivities, corresponding to Figure 3A (a-c) plots. H_2O: 5.0×10^{-4} ppm^{-1} (a); CO: 1.5×10^{-5} ppm^{-1} (b); 2.2×10^{-5} ppm^{-1} (c).

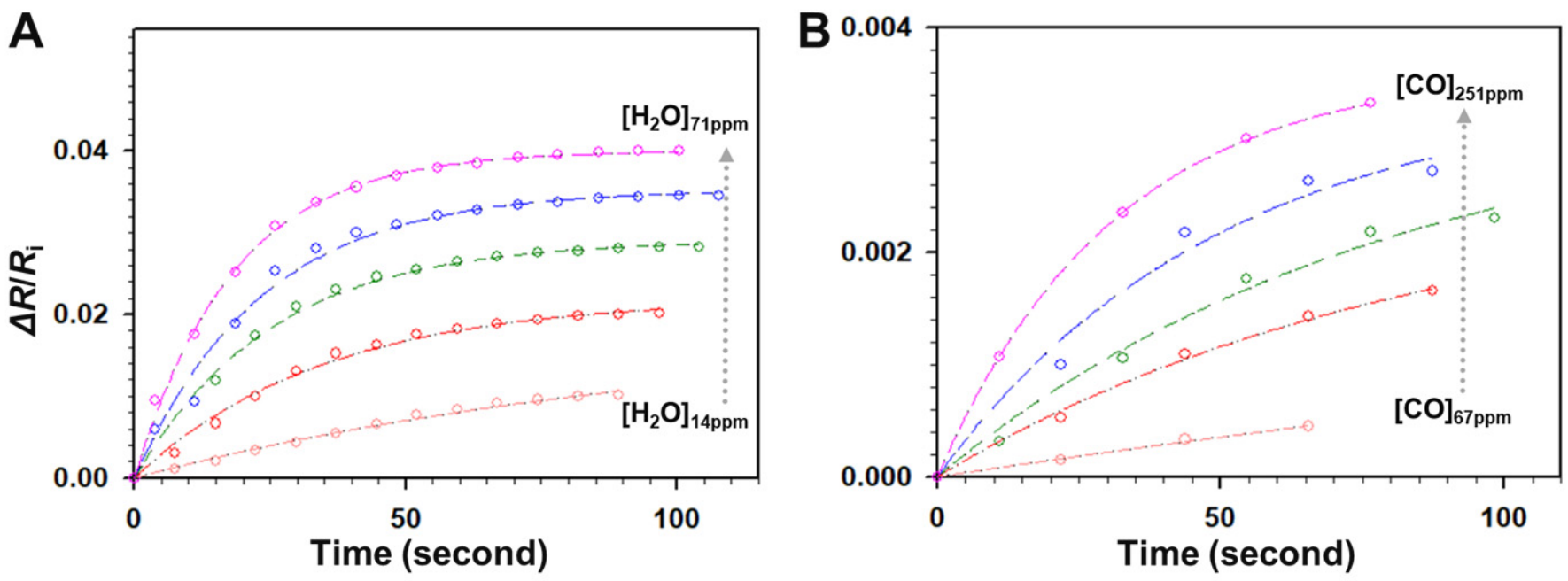

Figure S6A (corresponding to the response kinetics shown in Figure 3A(a))	$[H_2O]$/ppm	Fitting parameters *a*	*b*
	14	0.0183	0.0097
	28	0.022	0.0288
	43	0.0291	0.0396
	57	0.0353	0.0426
	71	0.04	0.0547

Figure S6B (corresponding to the response kinetics shown in Figure 3A(b))	[CO]/ppm	Fitting parameters *a*	*b*
	67	0.0026	0.0029
	113	0.0026	0.0117
	159	0.0027	0.0123
	205	0.0034	0.02
	251	0.0036	0.0317

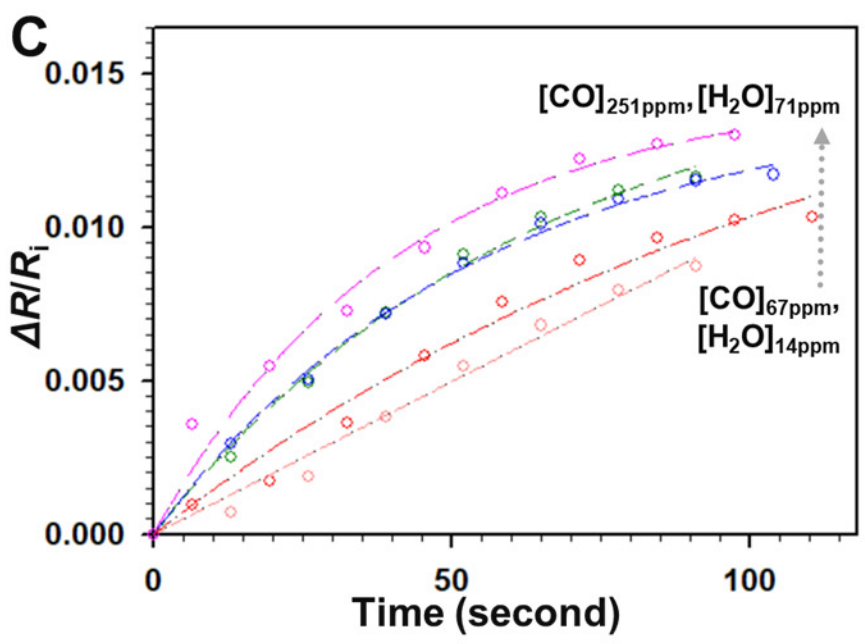

Figure S6C (corresponding to the response kinetics shown in Figure 3A(c))	[CO]/ ppm	$[H_2O]$/ ppm	Fitting parameters *a*	*b*
	67	14	1.1381	0.00009
	113	28	0.0186	0.0081
	159	43	0.0158	0.0155
	205	57	0.0141	0.0184
	251	71	0.0145	0.0243

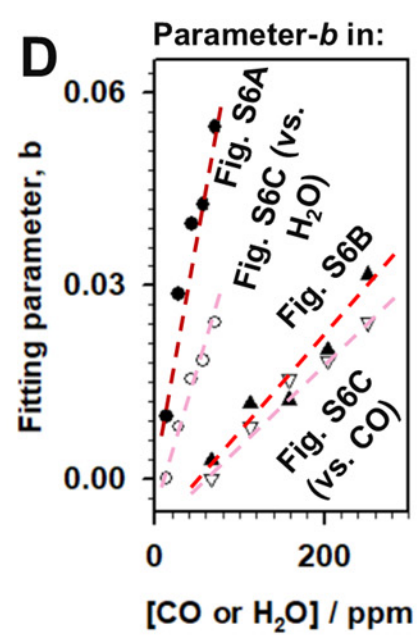

Figure S6. (A, B, C) Fitting to response kinetics in Figure 3A (a), (b), and (c), respectively. Dashed lines: the theoretical fit based on Langmuir adsorption isotherm. (D) Plots of values of *b* vs. CO or H_2O concentration (ppm) for A, B and C. Linear regression slope: (A) 7×10^{-4} s^{-1}, (B) 1×10^{-4} s^{-1}, and (C) 1×10^{-4} s^{-1} (vs. CO), and 4×10^{-4} s^{-1} (vs. H_2O).

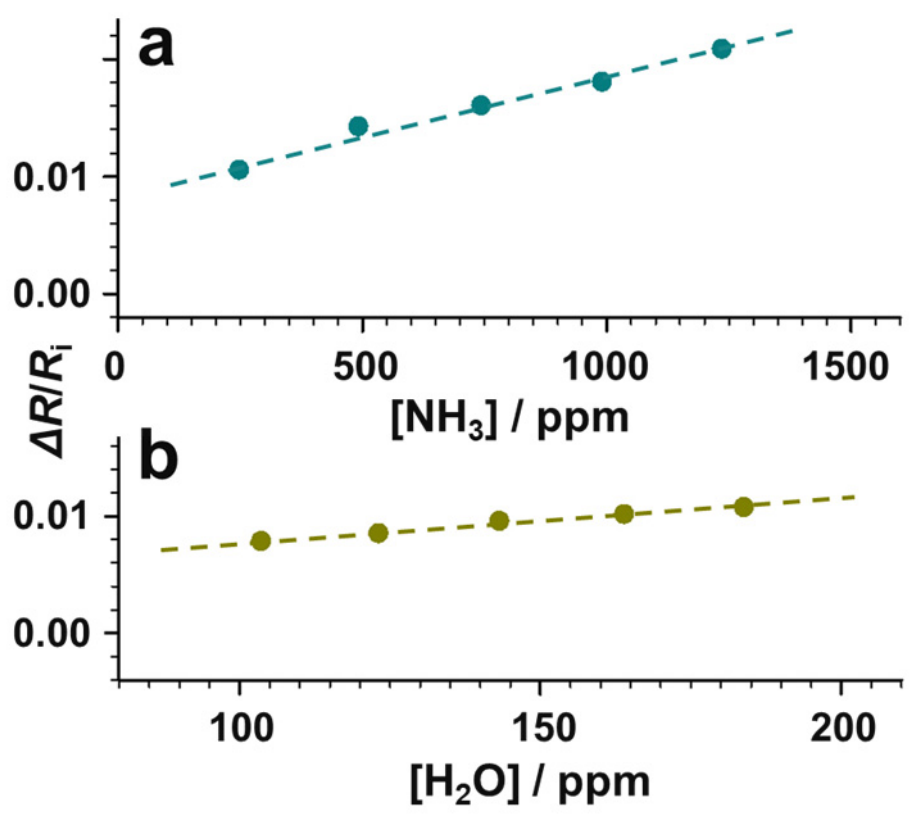

Figure S7. Response sensitivities, corresponding to Figure 4A (b-c) plots. (a) NH_3: 9.8×10^{-6} ppm^{-1} (LOD ~8.0 ppm). (b) H_2O: 3.7×10^{-5} ppm^{-1}.

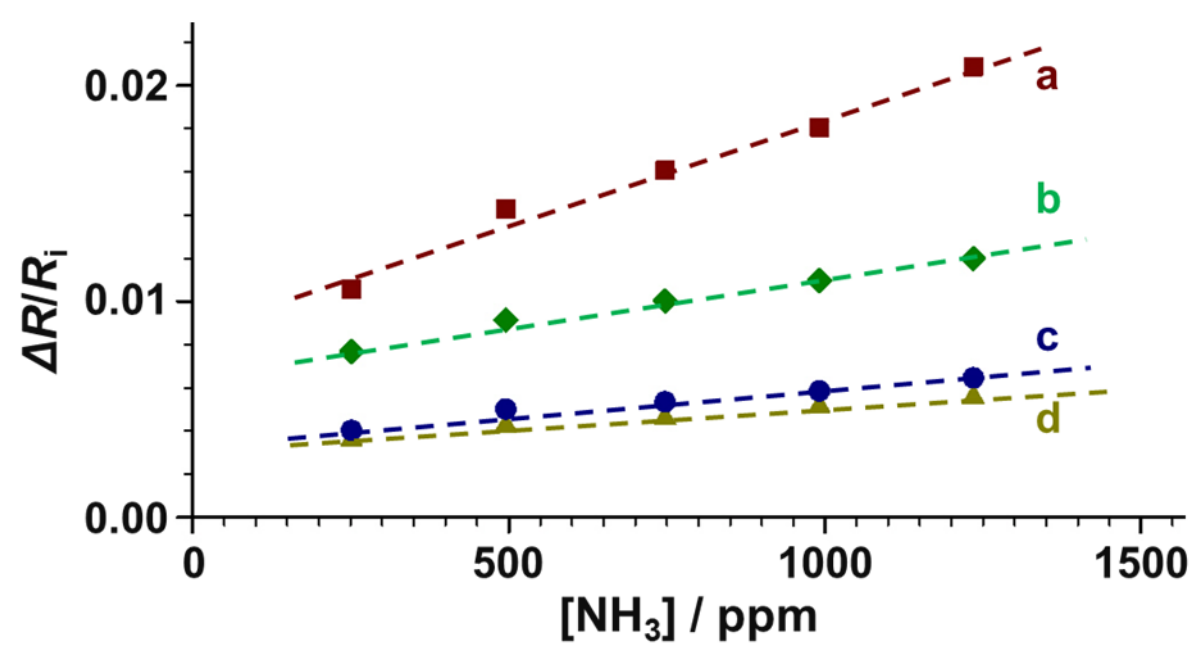

Figure S8. Comparison of the response profiles to mixtures of ammonia and water vapor with different concentrations for sensors in a sensor array of PDT-Au_{2nm} (a, 9.9×10^{-6} ppm^{-1}), NDT-Au_{2nm} (b, 4.2×10^{-6} ppm^{-1}), ODT-Au_{2nm} (c, 2.3×10^{-6} ppm^{-1}), and DDT-Au_{2nm} (d, 2.0×10^{-6} ppm^{-1}). Au_{2nm}: 2-nm sized Au NPs. The response sensitivities showed a subtle dependence on the linker chain length: DDT-Au_{2nm} (2.0×10^{-6} ppm^{-1}) < ODT-Au_{2nm} (2.3×10^{-6} ppm^{-1}) < NDT-Au_{2nm} (4.2×10^{-6} ppm^{-1}) < PDT-Au_{2nm} (9.9×10^{-6} ppm^{-1}).

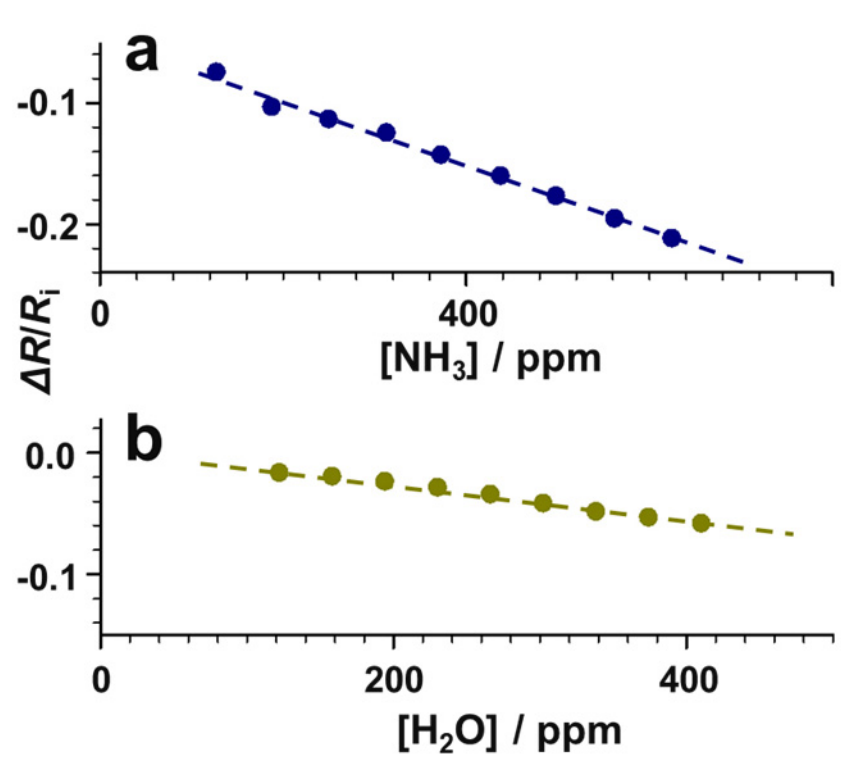

Figure S9. Response sensitivities, corresponding to Figure 4B (b-c) plots. (a) NH_3 sensitivity: -3.0×10^{-4} ppm^{-1} (LOD ~5.1 ppm); (b) water sensitivity: -2.0×10^{-4} ppm^{-1}.

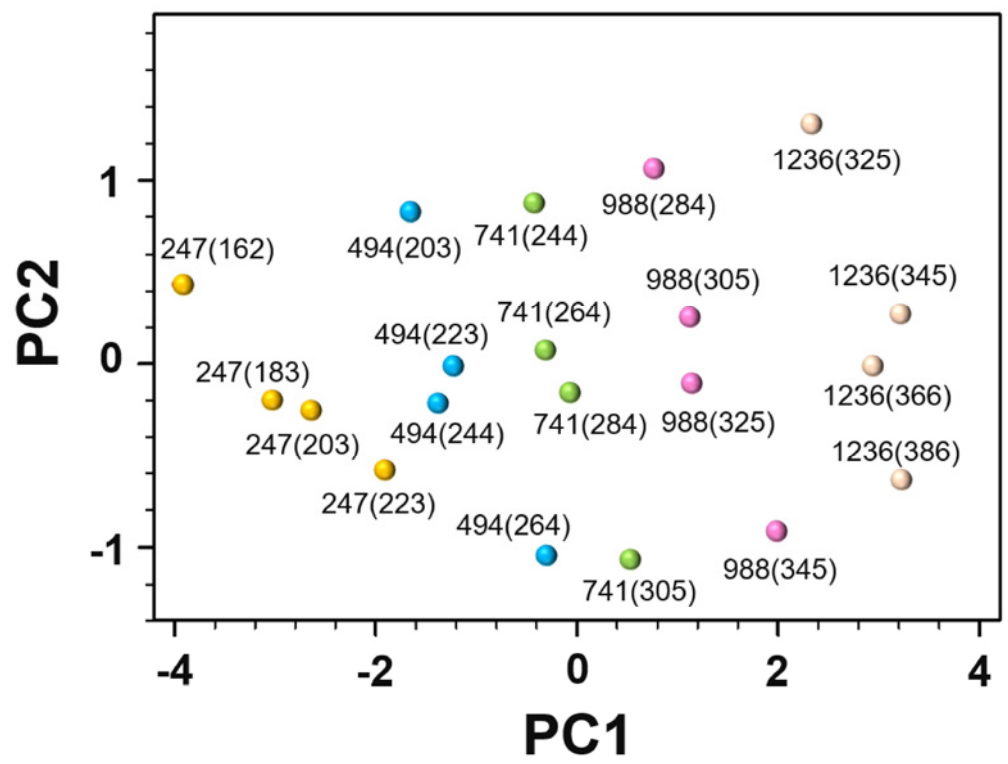

Figure S10. Principle component analysis (PCA) score plot for responses to NH_3 and H_2O vapor mixtures. The data label in the figure means the concentration (ppm) of components in the test mixture. For example, 247(162) means 247 ppm NH_3 and 162 ppm H_2O in the mixture.

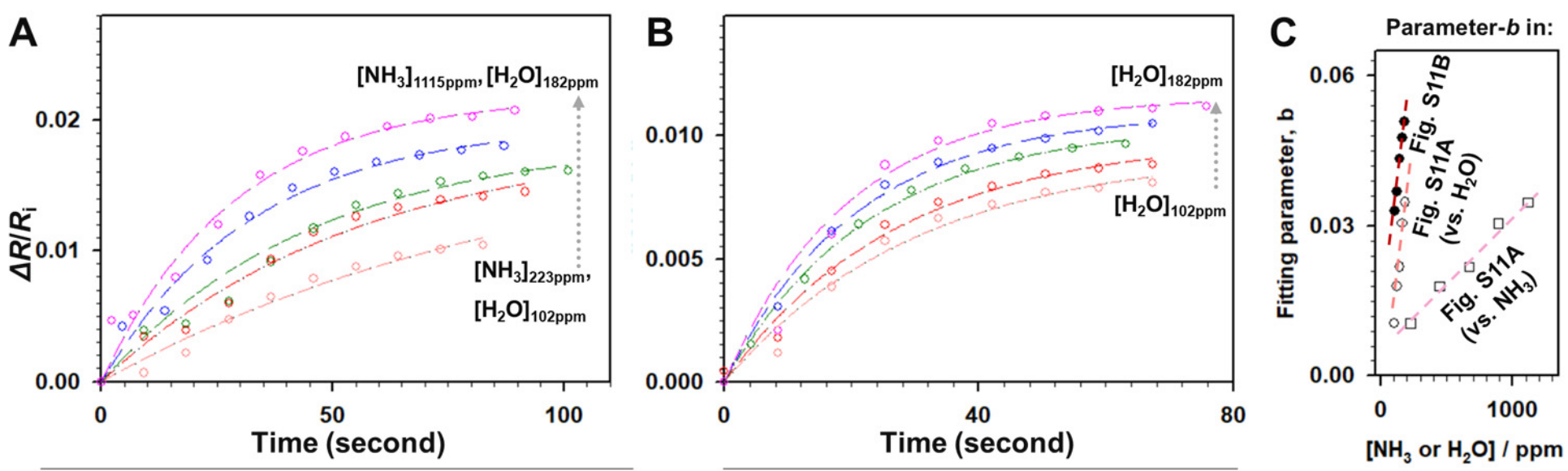

Figure S11A (corresponding to the response kinetics shown in Figure 4A(b))	$[NH_3]$/ ppm	$[H_2O]$/ ppm	Fitting parameters *a*	*b*
	223	102	0.0189	0.0105
	446	122	0.0188	0.0179
	669	142	0.0186	0.0218
	892	162	0.0198	0.0305
	1115	182	0.0219	0.0347

Figure S11B (corresponding to the response kinetics shown in Figure 4A(c))	$[H_2O]$/ ppm	Fitting parameters *a*	*b*
	102	0.0094	0.0329
	122	0.0099	0.0368
	142	0.0117	0.0434
	162	0.0105	0.0476
	182	0.0116	0.0508

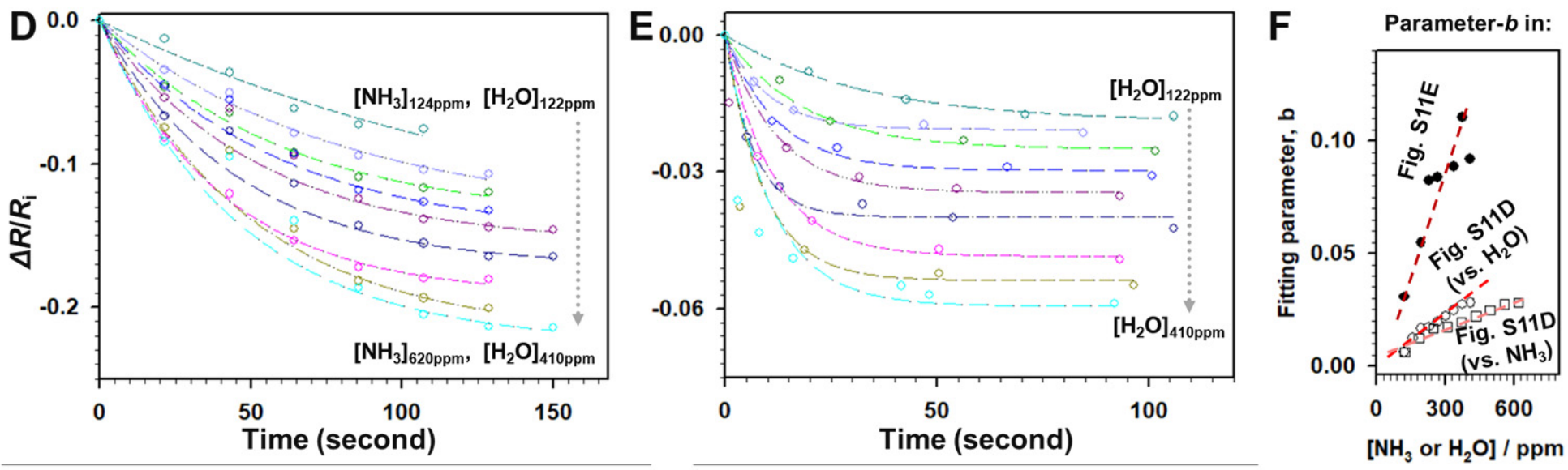

Figure S11D (corresponding to the response kinetics shown in Figure 4B(b))	$[NH_3]$/ ppm	$[H_2O]$/ ppm	Fitting parameters *a*	*b*
	124	122	-0.1647	0.0062
	186	158	-0.1389	0.0124
	248	194	-0.1384	0.0168
	310	230	-0.1498	0.0174
	372	266	-0.1557	0.0196
	434	302	-0.1719	0.0221
	496	338	-0.1918	0.0248
	558	374	-0.2189	0.0273
	620	410	-0.2253	0.0281

Figure S11E (corresponding to the response kinetics shown in Figure 4B(c))	$[H_2O]$/ ppm	Fitting parameters *a*	*b*
	122	-0.0190	0.0307
	158	-0.0207	0.0991
	194	-0.0249	0.0547
	230	-0.0296	0.0826
	266	-0.0345	0.0837
	302	-0.0399	0.1508
	338	-0.0485	0.0888
	374	-0.0538	0.1104
	410	-0.0594	0.0920

Figure S11. (A, B, D, E) Fitting to response kinetics in Figure 4A (b), 4A (c), 4B (b), and 4B (c), respectively. Dashed lines: the theoretical fit based on Langmuir adsorption isotherm. (C) Plots of values of *b* vs. NH_3 or H_2O concentration (ppm) for A and B. Linear regression slope: (A) 3×10^{-5} s^{-1} (vs. NH_3), and 3×10^{-4} s^{-1} (vs. H_2O), and (B) 2×10^{-4} s^{-1}. (F) Plots of values of *b* vs. NH_3 or H_2O concentration (ppm) for D and E. Linear regression slope: (D) 4×10^{-5} s^{-1} (vs. NH_3), and 7×10^{-5} s^{-1} (vs. H_2O), and (E) 2×10^{-4} s^{-1}.

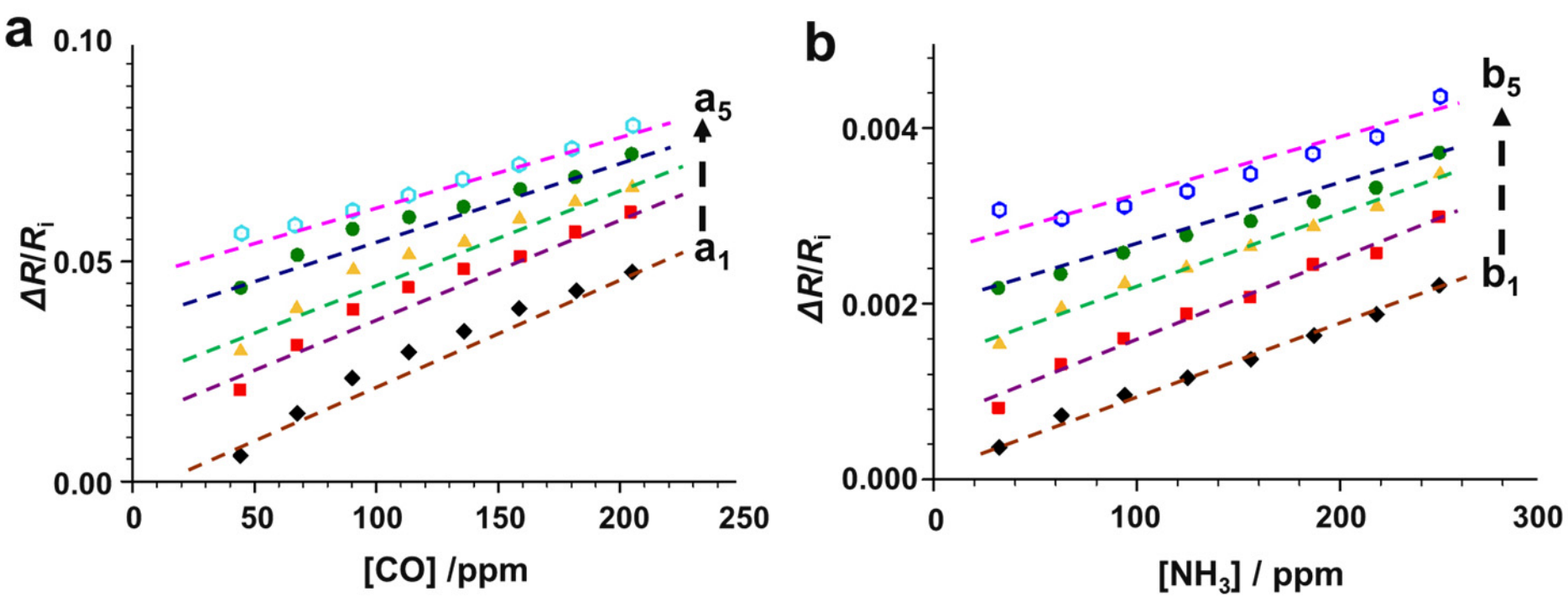

Figure S12. Response sensitivities, corresponding to Figure 5 (A-B) plots: (a) a_1: 2.5×10^{-4} (LOD ~0.3 ppm); a_2: 2.4×10^{-4} (LOD ~1.2 ppm); a_3: 2.2×10^{-4} (LOD ~0.6 ppm); a_4: 1.7×10^{-4} (LOD ~0.5 ppm); a_5: 1.6×10^{-4} (LOD ~1.1 ppm). (b) b_1: 1.8×10^{-4} (LOD ~0.1 ppm); b_2: 2.3×10^{-4} (LOD ~0.1 ppm); b_3: 1.7×10^{-4} (LOD ~0.1 ppm); b_4: 1.4×10^{-4} (LOD ~4.0 ppm); b_5: 1.3×10^{-4} (ppm^{-1}) (LOD ~3.6 ppm).

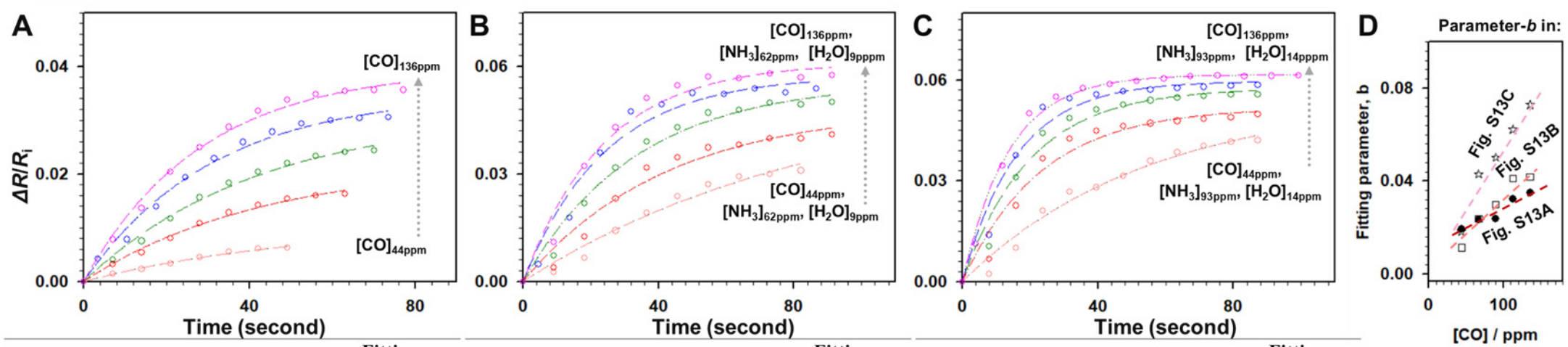

Figure S13A (corresponding to the response kinetics shown in Figure 5A(a_1))	[CO]/ ppm	[NH$_3$]/ ppm	[H$_2$O]/ ppm	Fitting parameters *a*	*b*
	44	0	0	0.0107	0.0192
	67	0	0	0.0220	0.0233
	90	0	0	0.0313	0.0236
	113	0	0	0.0384	0.0321
	136	0	0	0.0397	0.0350

Figure S13B (corresponding to the response kinetics shown in Figure 5A(a_3))	[CO]/ ppm	[NH$_3$]/ ppm	[H$_2$O]/ ppm	Fitting parameters *a*	*b*
	44	62	9	0.0543	0.0113
	67	62	9	0.0484	0.0237
	90	62	9	0.0556	0.0298
	113	62	9	0.0551	0.0412
	136	62	9	0.0611	0.0418

Figure S13C (corresponding to the response kinetics shown in Figure 5A(a_4))	[CO]/ ppm	[NH$_3$]/ ppm	[H$_2$O]/ ppm	Fitting parameters *a*	*b*
	44	93	14	0.0548	0.0182
	67	93	14	0.0519	0.0430
	90	93	14	0.0576	0.0502
	113	93	14	0.0597	0.0622
	136	93	14	0.0616	0.0729

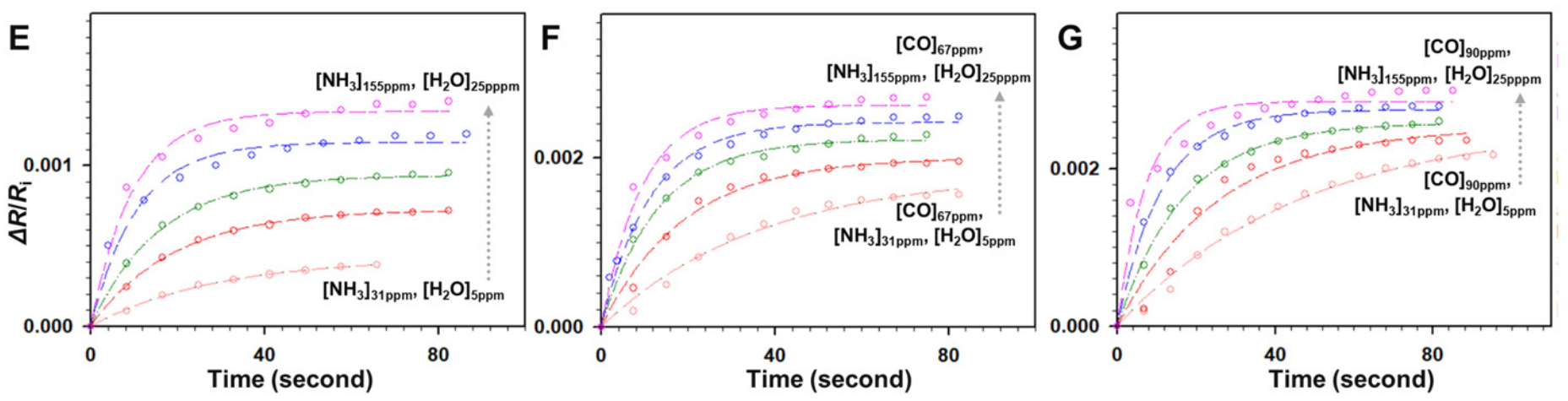

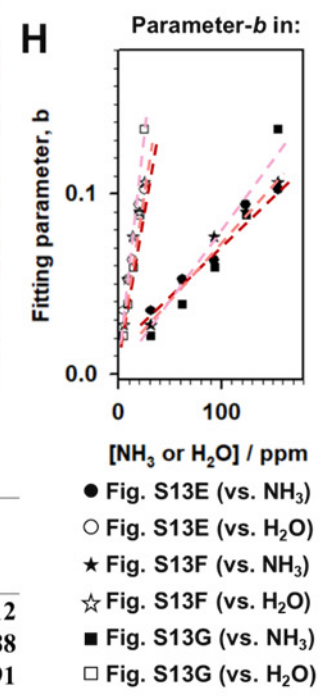

Figure S13E (corresponding to the response kinetics shown in Figure 5B(b_1))	[NH$_3$]/ ppm	[H$_2$O]/ ppm	[CO]/ ppm	Fitting parameters *a*	*b*
	31	5	0	0.0004	0.0351
	62	9	0	0.0007	0.0522
	93	14	0	0.0009	0.0635
	124	20	0	0.0011	0.0943
	155	25	0	0.0013	0.1029

Figure S13F (corresponding to the response kinetics shown in Figure 5B(b_3))	[NH$_3$]/ ppm	[H$_2$O]/ ppm	[CO]/ ppm	Fitting parameters *a*	*b*
	31	5	67	0.0018	0.027
	62	9	67	0.0020	0.0524
	93	14	67	0.0022	0.0763
	124	20	67	0.0024	0.0899
	155	25	67	0.0026	0.1066

Figure S13G (corresponding to the response kinetics shown in Figure 5B(b_4))	[NH$_3$]/ ppm	[H$_2$O]/ ppm	[CO]/ ppm	Fitting parameters *a*	*b*
	31	5	90	0.0026	0.0212
	62	9	90	0.0025	0.0388
	93	14	90	0.0026	0.0591
	124	20	90	0.0028	0.0882
	155	25	90	0.0029	0.1361

Figure S13. (A, B, C, E, F, G) Fitting to response kinetics in Figure 5A (a_1), 5A (a_3), 5A (a_4), 5B (b_1), 5B (b_3), and 5B (b_4), respectively. Dashed lines: the theoretical fit based on Langmuir adsorption isotherm. (D) Plots of values of *b* vs. CO concentration (ppm) for A, B and C. Linear regression slope: (A) 2×10^{-4} s^{-1} (vs. CO), (B) 3×10^{-4} s^{-1} (vs. CO), and (C) 6×10^{-4} s^{-1} (vs. CO). (H) Plots of values of *b* vs. NH_3 or H_2O concentration (ppm) for E, F and G. Linear regression slope: (E) 6×10^{-4} (vs. NH_3), 3.5×10^{-3} s^{-1} (vs. H_2O), (F) 6×10^{-4} s^{-1} (vs. NH_3), 3.8×10^{-3} s^{-1} (vs. H_2O), and (G) 9×10^{-4} s^{-1} (vs. NH_3), and 5.5×10^{-3} s^{-1} (vs. H_2O).

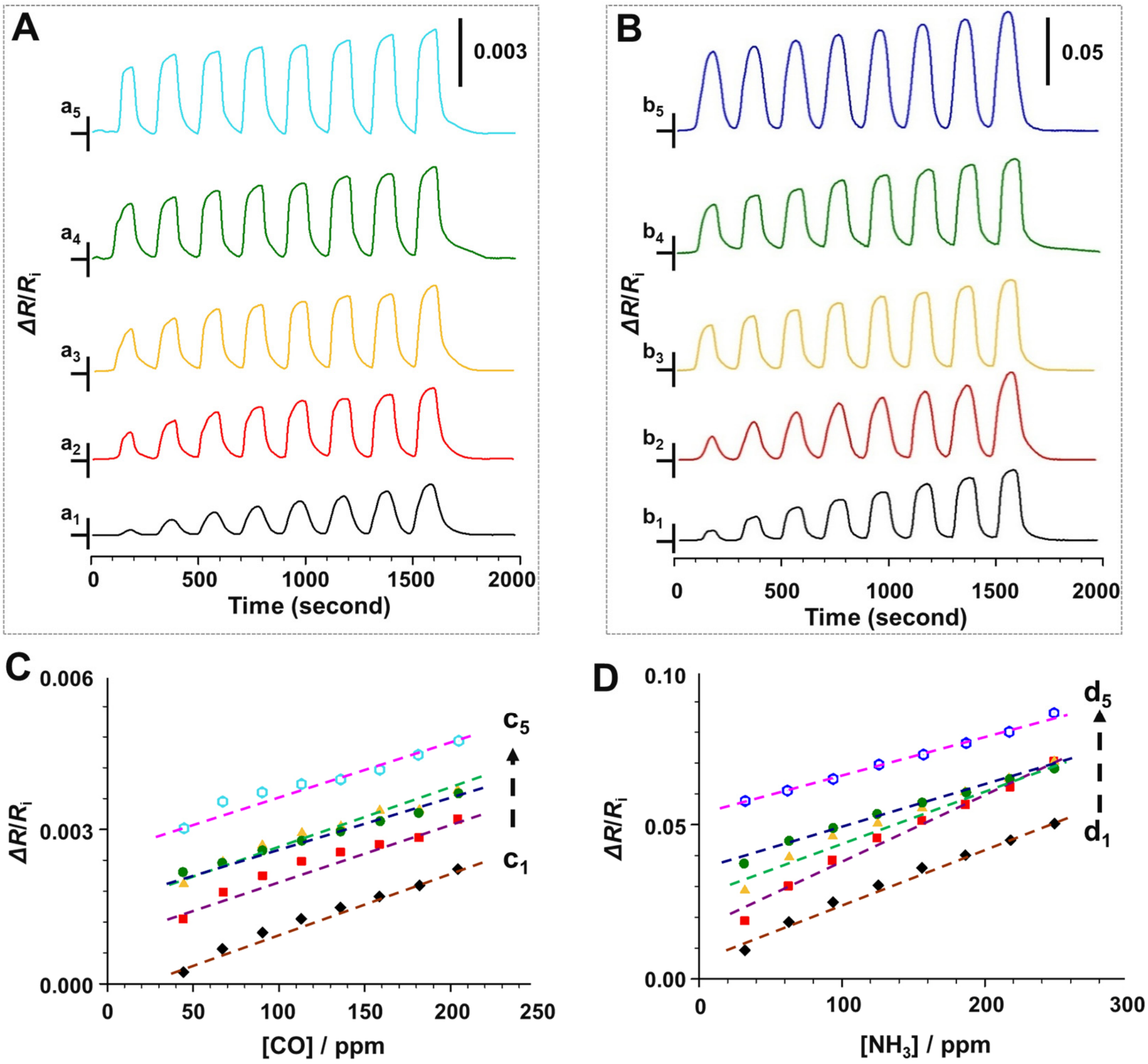

Figure S14. (a,c) Response profiles to ternary mixtures of CO, NH_3 and H_2O vapor by varying CO concentration (44, 67, 90, 113, 136, 159, 182, and 205 ppm) while keeping NH_3 and H_2O vapor at a series of constant concentrations (NH_3/H_2O concentration: a_1: 0/0 ppm, a_2: 31/5 ppm, a_3: 62/9 ppm, a_4: 93/14 ppm, and a_5: 185/27 ppm) for ODT-Au_{5nm} sensing films. (c) The corresponding response sensitivity plots. Slopes of linear regression: c_1: 1.2×10^{-5} (LOD ~0.6 ppm); c_2: 1.1×10^{-5} (LOD ~2.3 ppm c_3: 1.0×10^{-5} (LOD ~2.0 ppm); c_4: 9.2×10^{-6} (LOD ~6.8 ppm); c_5: 9.3×10^{-6} (LOD ~7.8 ppm). (b,d) Response profiles to ternary mixtures of CO, NH_3 and H_2O vapor by varying NH_3 and H_2O vapor concentration (NH_3/H_2O: 31/5, 62/9, 93/14, 124/20,155/25, 186/31, 217/36, and 248/41 ppm) while keeping CO at a series of constant concentrations (b_1: 0 ppm, b_2: 44 ppm, b_3: 67 ppm, b_4: 90 ppm, b_5: 158 ppm) for ODT-Au_{5nm} sensing films. (d) The corresponding response sensitivity plots. Slopes of linear regression (ppm^{-1}): d_1: 8.0×10^{-6}); d_2: 9.4×10^{-6} (LOD ~25.0 ppm); d_3: 8.4×10^{-6} (LOD ~20.6 ppm); d_4: 6.8×10^{-6}; d_5: 6.0×10^{-6}.

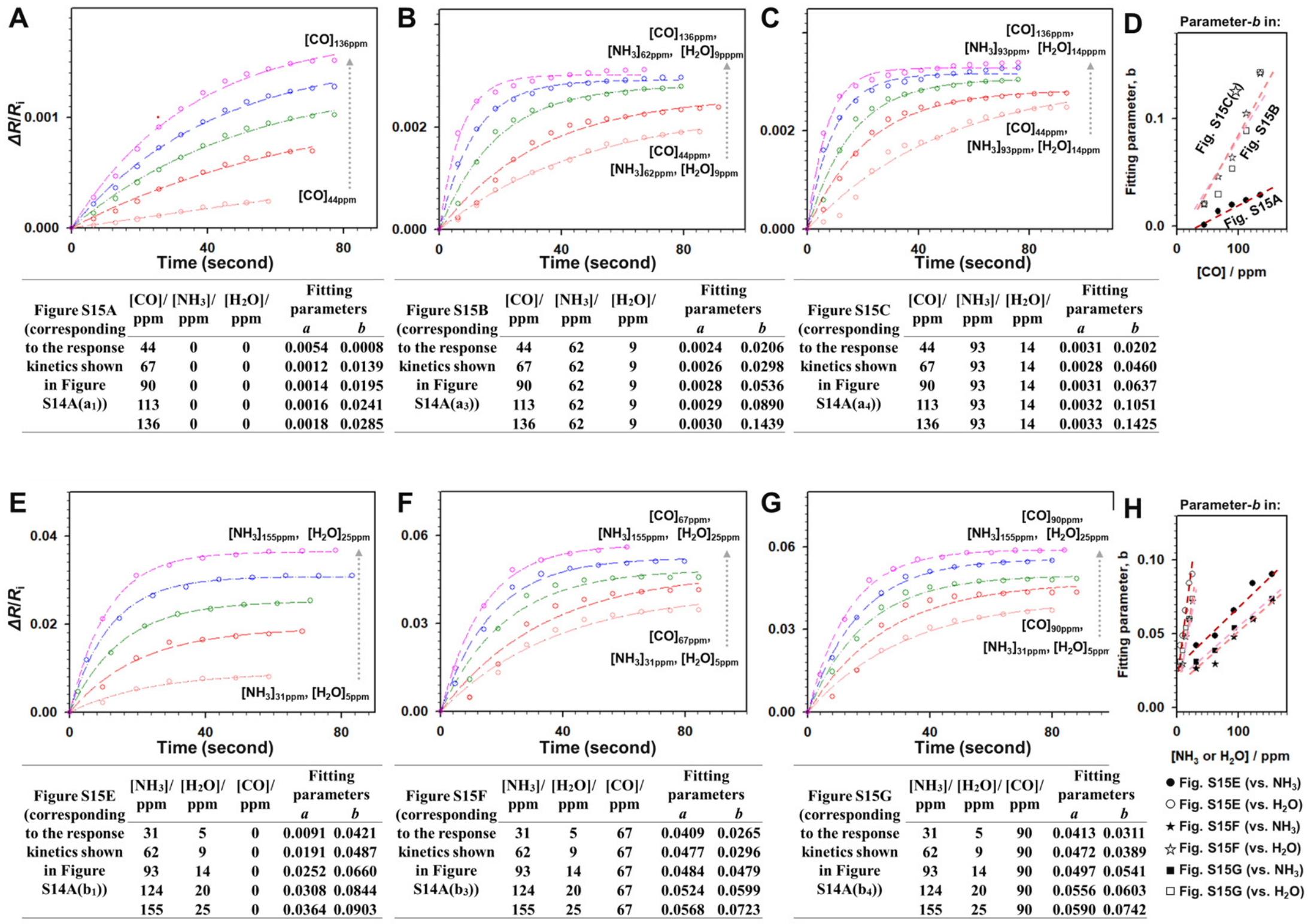

Figure S15A (corresponding to the response kinetics shown in Figure S14A(a_1))	[CO]/ ppm	[NH$_3$]/ ppm	[H$_2$O]/ ppm	Fitting parameters *a*	Fitting parameters *b*
	44	0	0	0.0054	0.0008
	67	0	0	0.0012	0.0139
	90	0	0	0.0014	0.0195
	113	0	0	0.0016	0.0241
	136	0	0	0.0018	0.0285

Figure S15B (corresponding to the response kinetics shown in Figure S14A(a_3))	[CO]/ ppm	[NH$_3$]/ ppm	[H$_2$O]/ ppm	Fitting parameters *a*	Fitting parameters *b*
	44	62	9	0.0024	0.0206
	67	62	9	0.0026	0.0298
	90	62	9	0.0028	0.0536
	113	62	9	0.0029	0.0890
	136	62	9	0.0030	0.1439

Figure S15C (corresponding to the response kinetics shown in Figure S14A(a_4))	[CO]/ ppm	[NH$_3$]/ ppm	[H$_2$O]/ ppm	Fitting parameters *a*	Fitting parameters *b*
	44	93	14	0.0031	0.0202
	67	93	14	0.0028	0.0460
	90	93	14	0.0031	0.0637
	113	93	14	0.0032	0.1051
	136	93	14	0.0033	0.1425

Figure S15E (corresponding to the response kinetics shown in Figure S14A(b_1))	[NH$_3$]/ ppm	[H$_2$O]/ ppm	[CO]/ ppm	Fitting parameters *a*	Fitting parameters *b*
	31	5	0	0.0091	0.0421
	62	9	0	0.0191	0.0487
	93	14	0	0.0252	0.0660
	124	20	0	0.0308	0.0844
	155	25	0	0.0364	0.0903

Figure S15F (corresponding to the response kinetics shown in Figure S14A(b_3))	[NH$_3$]/ ppm	[H$_2$O]/ ppm	[CO]/ ppm	Fitting parameters *a*	Fitting parameters *b*
	31	5	67	0.0409	0.0265
	62	9	67	0.0477	0.0296
	93	14	67	0.0484	0.0479
	124	20	67	0.0524	0.0599
	155	25	67	0.0568	0.0723

Figure S15G (corresponding to the response kinetics shown in Figure S14A(b_4))	[NH$_3$]/ ppm	[H$_2$O]/ ppm	[CO]/ ppm	Fitting parameters *a*	Fitting parameters *b*
	31	5	90	0.0413	0.0311
	62	9	90	0.0472	0.0389
	93	14	90	0.0497	0.0541
	124	20	90	0.0556	0.0603
	155	25	90	0.0590	0.0742

Figure. S15. (A, B, C, E, F, G) Fitting to response kinetics in Figure S14A (a_1), S14A (a_3), S14A (a_4), S14B (b_1), S14B (b_3), and S14B (b_4), respectively. Dashed lines: the theoretical fit based on Langmuir adsorption isotherm. (D) Plots of values of *b* vs. CO concentration (ppm) for A, B and C. Linear regression slope: (A) 3×10^{-4} s^{-1} (vs. CO), (B) 1.3×10^{-3} s^{-1} (vs. CO), and (C) 1.3×10^{-3} s^{-1} (vs. CO). (H) Plots of values of *b* vs. NH_3 or H_2O concentration (ppm) for E, F and G. Linear regression slope: (E) 4×10^{-4} s^{-1} (vs. NH_3), 2.6×10^{-3} s^{-1} (vs. H_2O), (F) 4×10^{-4} s^{-1} (vs. NH_3), 2.4×10^{-3} s^{-1} (vs. H_2O), and (G) 3×10^{-4} s^{-1} (vs. NH_3), and 2.1×10^{-3} s^{-1} (vs. H_2O).

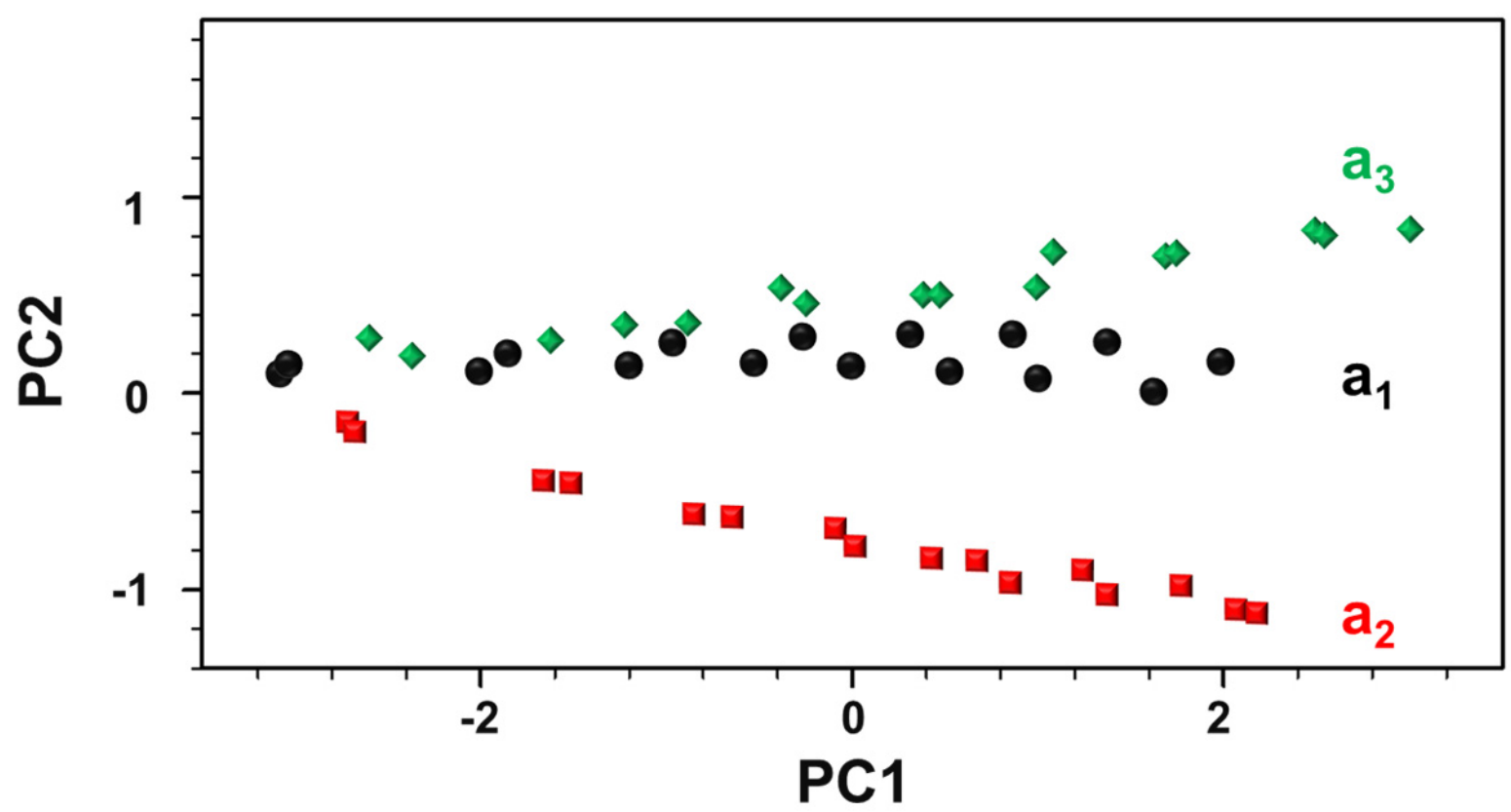

Figure S16. PCA score plot for CO (a_1), H_2O vapor (a_2), and NH_3 + H_2O vapor mixture (a_3), respectively. Sensor array: PDT-Au_{2nm}, PDT-Au_{5nm}, and ODT-Au_{2nm} films.

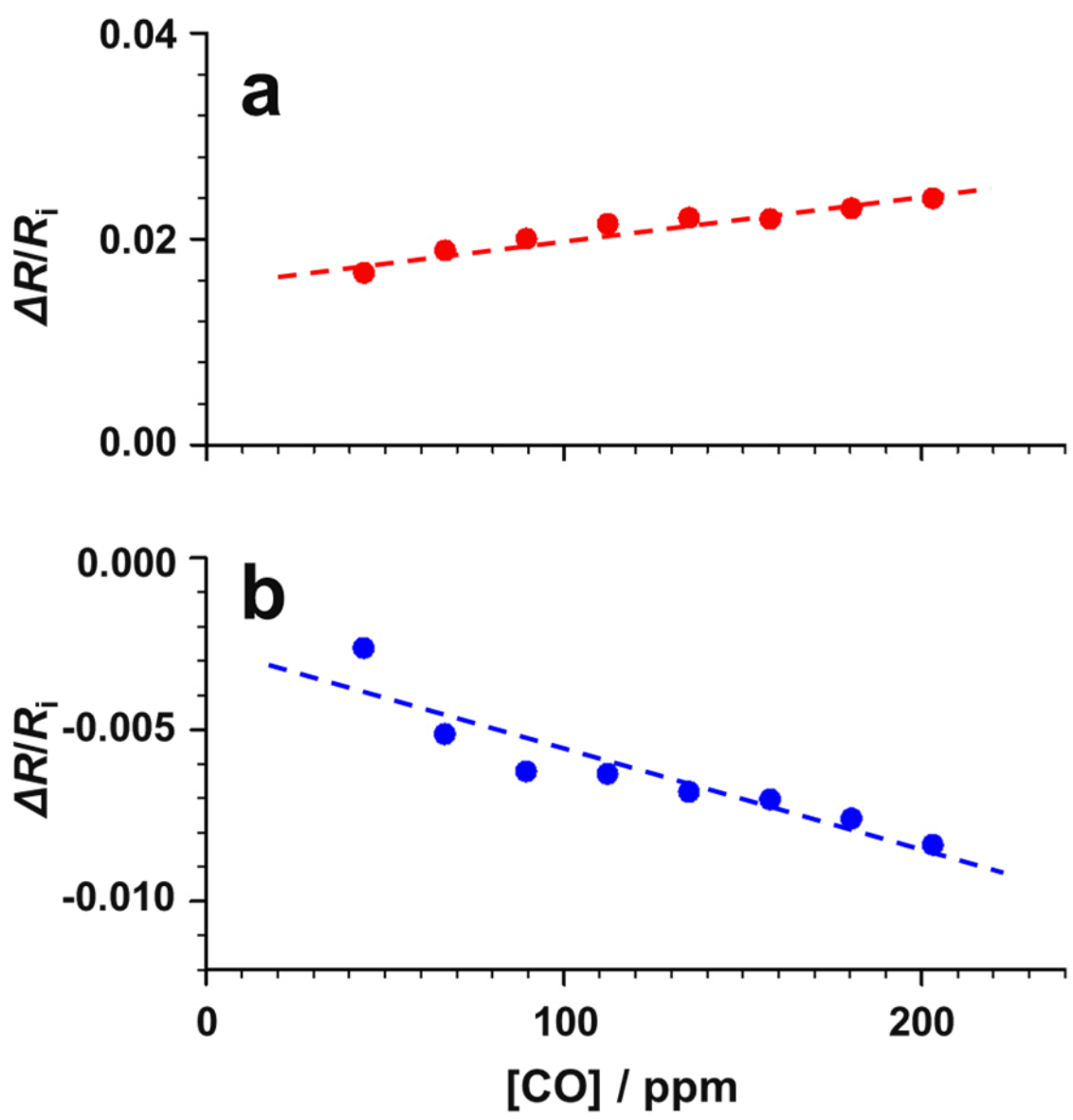

Figure S17. Response sensitivities, corresponding to Figure 6 (a-b) plots. (a) CO: 4.1×10^{-5} ppm^{-1}; (b): CO: -2.9×10^{-5} ppm^{-1}.

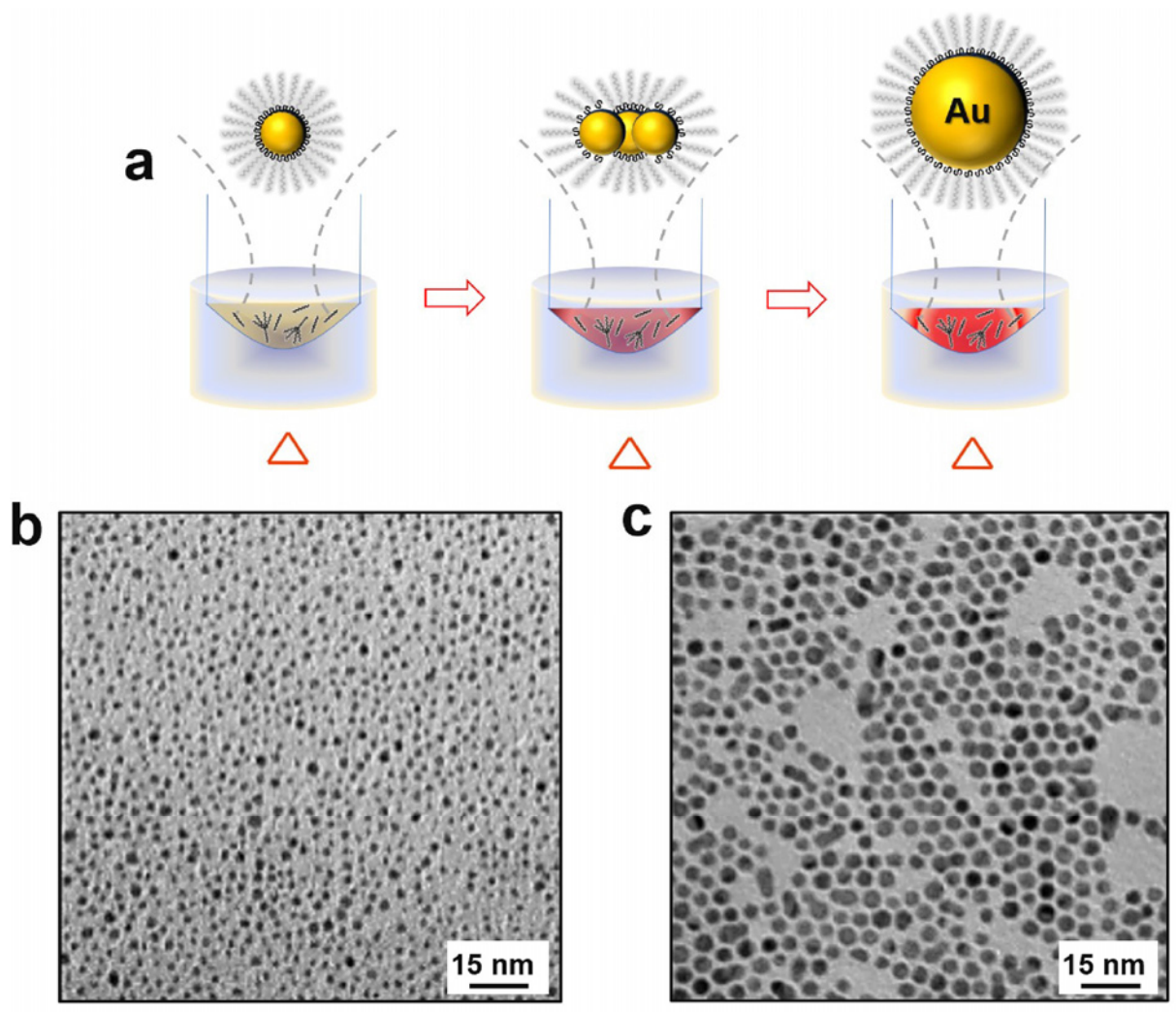

Figure S18. (a) Schematic illustration of the thermally activated evolution process from two-phase synthesized smaller-sized (e.g., ~2 nm) gold nanoparticles encapsulated with decanethiolate (DT) monolayer shells (Au_{2nm}-DT) to larger-sized nanoparticles (e.g., ~5 nm) [27]. (b, c) TEM images of the as-synthesized gold nanoparticles featuring 1.9 ±0.5 nm (b) and 4.6±0.6 nm (c) diameters. Note that capping molecules are not to scale and represent an idealized (defect-free) monolayer packing on the nanoparticle surface.

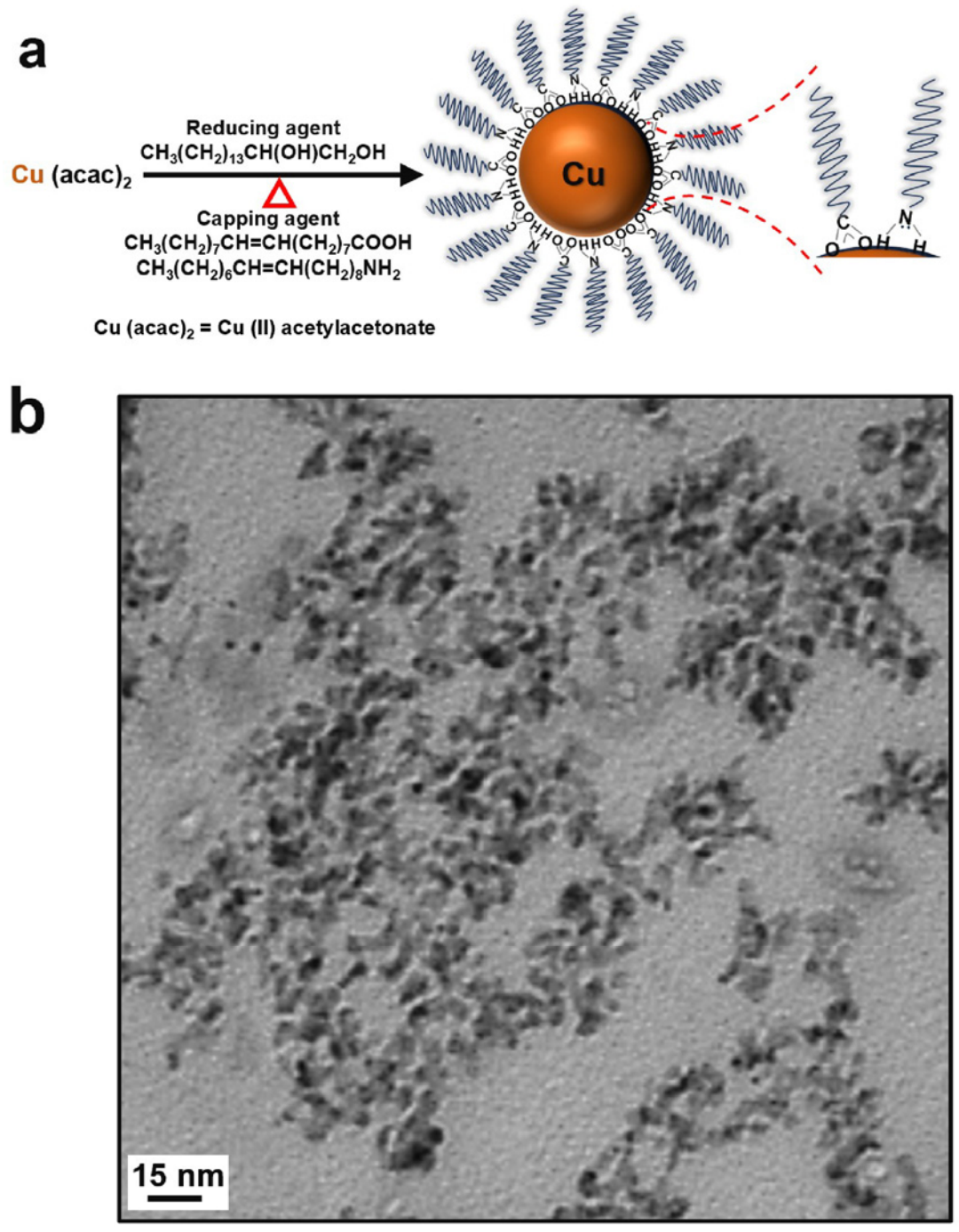

Figure S19. (a) Schematic illustration of the chemical reduction of copper nanoparticles at elevated temperature [28]. (b) TEM of copper nanoparticles capped with a shell of oleic acid and oleyl amine synthesized at 150 ºC (average size: 4.6 ±1.2 nm). Note that capping molecules are not to scale and represent an idealized (defect-free) monolayer packing on the nanoparticle surface.

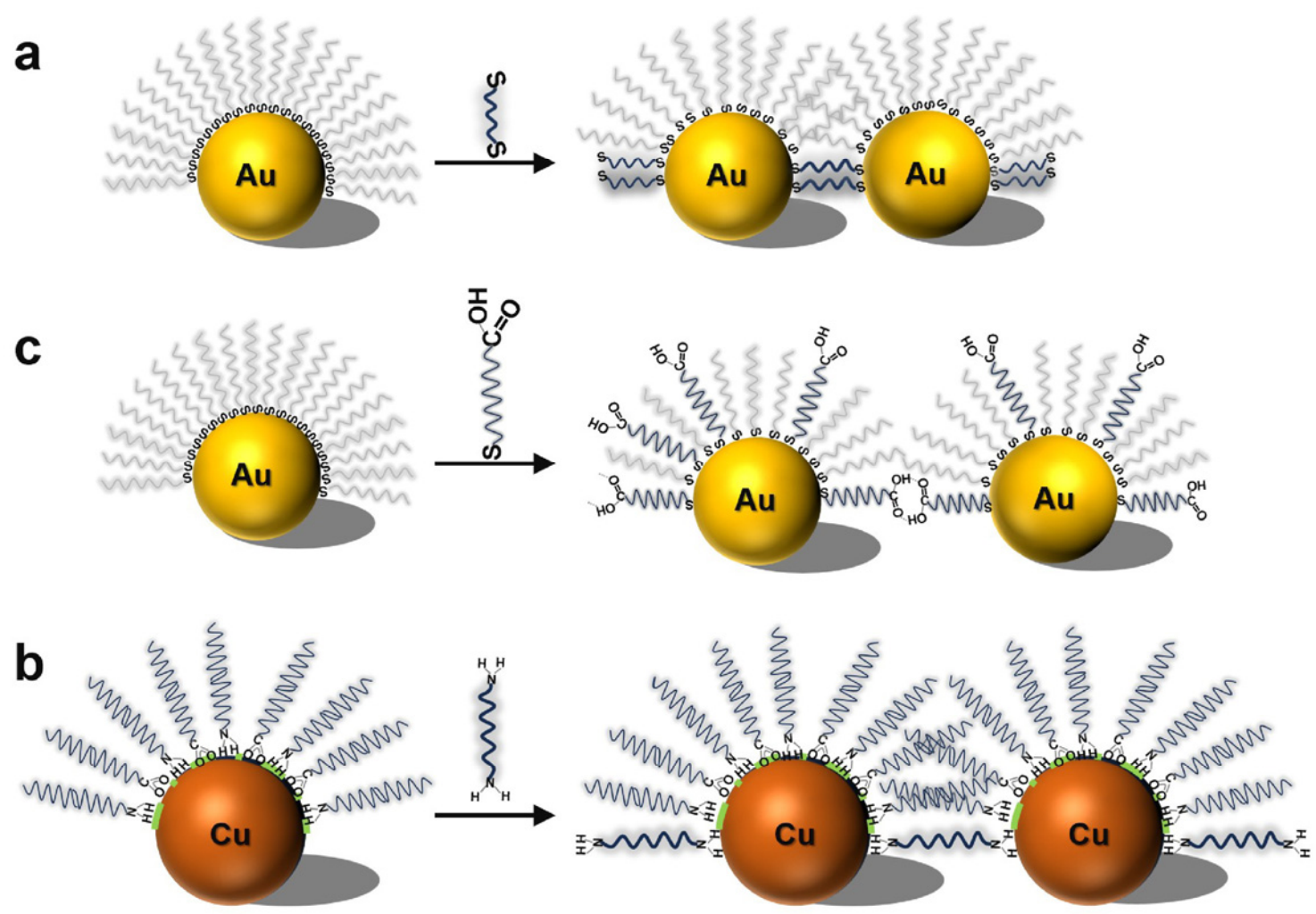

Figure S20. (a, b, c) Schematic illustration of idealized capping and linking molecules in the molecularly mediated thin film assembly of Au (a, b) and Cu NPs (c) using bifunctional linker molecules, alkyl dithiols (e.g., 1,5-pentanedithiol) (a), carboxylic functionalized alkyl thiols (e.g., 11-mercaptoundecanoic acid) (b), and alkyl diamine (e.g., 1,8-octanediamine) (c). Note that capping and linking molecules are shown on a portion of the nanoparticles and are not to scale; they depict an idealized (defect-free) monolayer packing on the surface.

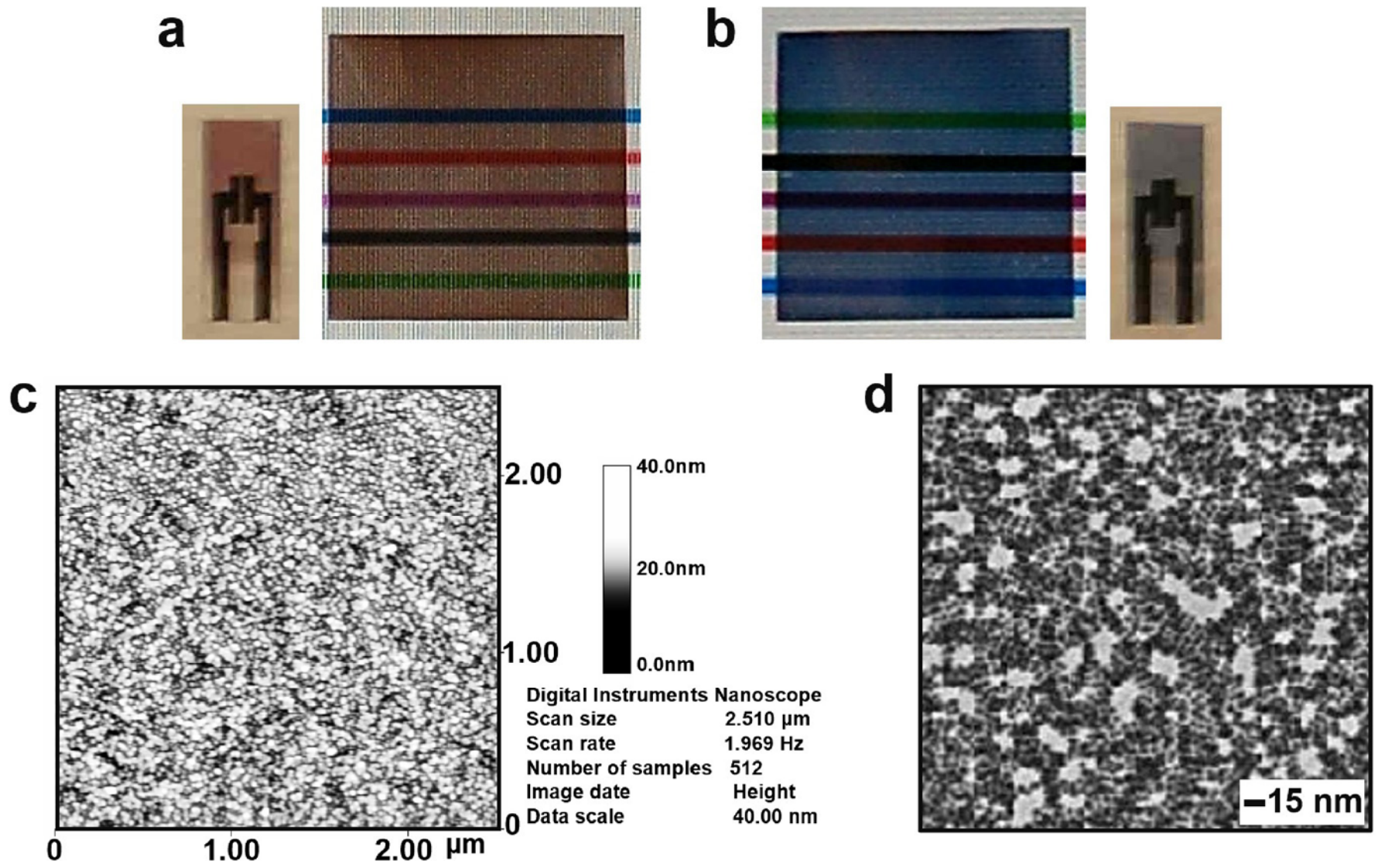

Figure S21. (a-b) Photos showing thin films on glass substrates which are placed on top of colored stripes to illustrate the thin film transparency and on IME/glass devices: (a) NDT-Au$_{nm}$ and (b) MUA-Au$_{nm}$ sensing films. (c-d) Representative AFM (c) and TEM (d) images of the thin film assemblies of nanoparticles. AFM/TEM characterizations of related thin film assemblies were described previously [6, 29].

References:

[1] M. J. Hostetler, J. E. Wingate, C. J. Zhong, J. E. Harris, R. W. Vachet, M. R. Clark, J. D. Londono, S. J. Green, J. J. Stokes, G. D. Wignall, G. L. Glish, M. D. Porter, N. D. Evans, R. W. Murray, "Alkanethiolate Gold Cluster Molecules with Core Diameters from 1.5 to 5.2 nm: Core and Monolayer Properties as a Function of Core Size," *Langmuir* 14 (1998): 17–30. https://doi.org/10.1021/la970588w

[2] M. M. Maye, W. X. Zheng, F. L. Leibowitz, N. K. Ly, C. J. Zhong, "Heating-Induced Evolution of Thiolate-Encapsulated Gold Nanoparticles: A Strategy for Size and Shape Manipulations," *Langmuir* 16 (2000): 490–497. https://doi.org/10.1021/la990892k

[3] D. Mott, J. Galkowski, L. Wang, J. Luo, C. J. Zhong, "Synthesis of Size-Controlled and Shaped Copper Nanoparticles," *Langmuir* 23 (2007): 5740–5745. https://doi.org/10.1021/la0635092

[4] D. Mott, J. Yin, M. Engelhard, R. Loukrakpam, P. Chang, G. Miller, I.-T. Bae, N. Chandra Das, C. Wang, J. Luo, C. J. Zhong, "From Ultrafine Thiolate-Capped Copper Nanoclusters toward Copper Sulfide Nanodiscs: A Thermally-Activated Evolution Route," *Chem. Mater*. 22 (2010): 261–271. https://doi.org/10.1021/cm903038w

[5] F. L. Leibowitz, W. X. Zheng, M. M. Maye, C. J. Zhong, "Structures and Properties of Nanoparticle Thin Films Formed via a One-Step Exchange-Crosslinking-Precipitation Route," *Anal. Chem.* 71 (1999): 5076–5083. https://doi.org/10.1021/ac990752f

[6] L. Han, J. Luo, N. Kariuki, M. M. Maye, V.W. Jones, C. J. Zhong, "Novel Interparticle Spatial Properties of Hydrogen-Bonding Mediated Nanoparticle Assembly," *Chem. Mater.* 15 (2003): 29–37. https://doi.org/10.1021/cm0208051

[7] N. N. Kariuki, J. Luo, A. Hassan, I. S. Lim, L. Wang, C. J. Zhong, "Assembly of Bimetallic Gold-Silver Nanoparticles via Selective Interparticle Dicarboxylate-Silver Linkages," *Chem. Mater.* 18 (2006): 123–132. https://doi.org/10.1021/cm0519479

[8] L. Han, M. M. Maye, F. L. Leibowitz, N. K. Ly, C. J. Zhong, "Quartz-Crystal Microbalance and Spectrophotometric Assessments of Inter-Core and Inter-Shell Reactivities in Nanoparticle Film Formation and Growth," *J. Mater. Chem*. 11(2001): 1258–1264. https://doi.org/10.1039/b006687g

[9] L. Y. Wang, D. Miller, Q. Fan, J. Luo, M. Schadt, Q. Rendeng, G. R. Wang, J. Wang, G. R. Kowach, C. J. Zhong, "Assessment of Morphological and Optical Properties of Molecularly-Mediated Thin Film Assembly of Gold Nanoparticles," *J. Phys. Chem., C* 112 (2008): 2448–2455. https://doi.org/10.1021/jp710095r

[10] L. Wang, X. Shi, N. N. Kariuki, M. Schadt, G. R. Wang, Q. Rendeng, J. Choi, J. Luo, S. Lu, C. J. Zhong, "Array of Molecularly-Mediated Thin Film Assemblies of Nanoparticles: Correlation of Vapor Sensing with Interparticle Spatial Properties," *J. Am. Chem. Soc*. 129 (2007): 2161–2170. https://doi.org/10.1021/ja0673074

[11] G. R. Wang, L. Wang, Q. Rendeng, J. Wang, J. Luo, C. J. Zhong, "Correlation between Nanostructural Parameters and Conductivity Properties for Thin Film Assemblies of Gold Nanoparticles," *J. Mater. Chem.* 17 (2007): 457–462. https://doi.org/10.1039/b614366k

[12] S. Lim, C. J. Zhong, "Molecularly-Mediated Processing and Assembly of Nanoparticles: Exploring the Interparticle Interactions and Structures," *Acc. Chem. Res.* 42 (2009): 798–808. https://doi.org/10.1021/ar8002688

[13] J. Yin, P. Hu, J. Luo, L. Wang, M. F. Cohen, C. J. Zhong, "Molecularly–Mediated Thin Film Assembly of Nanoparticles on Flexible Devices: Electrical Conductivity vs. Device Strains in

Different Gas/Vapor Environment," *ACS Nano* 5 (2011): 6516–6526. https://doi.org/10.1021/nn201858c

[14] L. Wang, N. N. Kariuki, M. Schadt, D. Mott, J. Luo, X. Shi, C. Zhang, W. Hao, S. Lu, N. Kim, J.-Q. Wang, C. J. Zhong, "Sensing Arrays Constructed from Nanoparticle Thin Films and Interdigitated Microelectrodes," *Sensors* 6 (2006): 667–679. https://doi.org/10.3390/s6060667

[15] K. Alzoubi, M. M. Hamasha, L. Wang, H. Zhang, J. Yin, J. Luo, S. Lu, B. Sammakia, M. Poliks, C. J. Zhong, "Stability of Interdigitated Microelectrodes of Flexible Chemiresistor Sensors," *J. Display Technology* 8 (2012): 377-384. https://doi.org/10.1109/JDT.2012.2186953

[16] L. Han, D. R. Daniel, M. M. Maye, C. J. Zhong, "Core-Shell Nanostructured Nanoparticle Films as Chemically-Sensitive Interfaces," *Anal. Chem*. 73 (2001): 4441–4449. https://doi.org/10.1021/ac0104025

[17] W. Zhao, L. F. Al-Nasser, S. Shan, J. Li, Z. Skeete, N. Kang, J. Luo, S. Lu, C. J. Zhong, R. Harris, C. J. Grausgruber, "Detection of Mixed Volatile Organic Compounds and Lung Cancer Breaths Using Chemiresistor Arrays with Crosslinked Nanoparticle Thin Films," *Sensors Actuators, B.* 232 (2016): 292–299. http://dx.doi.org/10.1016/j.snb.2016.03.121

[18] Z. Xu, X. Shi, L. Wang, J. Luo, C. J. Zhong, S. Lu, "Pattern Recognition for Sensor Array Signals Using Fuzzy ARTMAP," *Sensors Actuators, B.* 141 (2009): 458–464. http://dx.doi.org/10.1016/j.snb.2009.06.046

[19] J. Luo, J. Luo, L. Wang, X. Shi, J. Yin, E. Crew, S. Lu, L. M. Lesperance, C. J. Zhong, "Nanoparticle-Structured Thin Film Sensor Arrays for Breath Sensing," *Sensors Actuators, B.* 161 (2012): 845–854. http://dx.doi.org/10.1016/j.snb.2011.11.045

[20] D. Dinh, G. Shang, L. Cai, W. B. Chan, A. Ma, J. Li, A. Huang, M. E. Louis, F. Cen, J. E. Halim, J. Guo, L. Gebre, Q. Liu, Z. Li, L. Yang, J. Luo, S. Lu, C. J. Zhong, "Artificial Intelligence-Enhanced Optimization of Wireless Breath Sensor Arrays for Detection of Lung Cancer Using Fuzzy Logic-Guided Genetic Algorithm and Multimodal Machine Learning," *ACS Sensors* 11 (2026): 2520–2531. https://doi.org/10.1021/acssensors.5c04441

[21] J. Luo, M. M. Maye, L. Han, N. Kariuki, V. W. Jones, Y. Lin, M. H. Engelhard, C. J. Zhong, "Spectroscopic Characterizations of Molecularly-Linked Gold Nanoparticle Assemblies Upon Thermal Treatment," *Langmuir* 20 (2004): 4254–4260. https://doi.org/10.1021/la036163t

[22] S. Shan, W. Zhao, J. Luo, J. Yin, J. C. Switzer, P. Joeseph, S. Lu, M. Poliks, and C. J. Zhong, "Flexibility Characteristics of Polyethylene Terephthalate Chemiresistor Coated with a Nanoparticle Thin Film Assembly," *J. Mater. Chem. C* 2 (2014): 1893–1903. https://doi.org/10.1039/c3tc32332c

[23] W. Zhao, J. Luo, S. Shan, J. P. Lombardi, Y. Xu, K. Cartwright, S. Lu, M. Poliks, C. J. Zhong, "Nanoparticle-Structured Highly Sensitive and Anisotropic Gauge Sensors," *Small* 35 (2015): 4509–4516. https://doi.org/10.1002/smll.201500768

[24] G. Shang, D. Dinh, T. Mercer, S. Yan, S. Wang, B. Malaei, J. Luo, S. Lu, C. J. Zhong, "Chemiresistive Sensor Array with Nanostructured Interfaces for Detection of Human Breaths with Simulated Lung Cancer Breath VOCs," *ACS Sensors* 8 (2023): 1328–1338. https://doi.org/10.1021/acssensors.2c02839

[25] S. Yan, S. Shan, J. Wen, J. Li, N. Kang, Z. Wu, J. Lombardi, H. W. Cheng, J. Wang, J. Luo, N. He, D. Mott, L. Wang, Q. Ge, B. S. Hsiao, M. Poliks, C. J. Zhong, "Surface-Mediated Interconnections of Nanoparticles in Cellulosic Fibrous Materials Towards 3D Sensors," *Adv. Mater.* 32 (2020):

2002171. https://doi.org/10.1002/adma.202002171

[26] H. W. Cheng, Z. P. Wu, S. Yan, J. Li, S. Shan, L. Wang, M. D. Porter, C. J. Zhong, "A Simple Vaporous Probe with Atomic-Scale Sensitivity to Structural Ordering and Orientation of Molecular Assembly," *Chem. Sci*. 10 (2019): 7104–7110. https://doi.org/10.1039/c9sc01656b

[27] H. W. Cheng, M. J. Schadt, C. J. Zhong, "Assessing the Role of Capping Molecules in Controlling Aggregative Growth of Gold Nanoparticles in Heated Solution," *Chem. Asian J*. 11, (2016): 120–127. https://doi.org/10.1002/asia.201500857

[28] D. Mott, J. Galkowski, L. Wang, J. Luo, C. J. Zhong, Synthesis of Size-Controlled and Shaped Copper Nanoparticles," *Langmuir* 23 (2007): 5740–5745. https://doi.org/10.1021/la0635092

[29] L. Wang, J. Luo, M. J. Schadt, C. J. Zhong. "Thin Film Assemblies of Molecularly-Linked Metal Nanoparticles and Multifunctional Properties," *Langmuir* 26 (2010): 618–632. https://doi.org/10.1021/la901811g
